# Renal cancer: new models and approach for personalizing therapy

**DOI:** 10.1186/s13046-018-0874-4

**Published:** 2018-09-05

**Authors:** Simona di Martino, Gabriele De Luca, Ludovica Grassi, Giulia Federici, Romina Alfonsi, Michele Signore, Antonio Addario, Laura De Salvo, Federica Francescangeli, Massimo Sanchez, Valentina Tirelli, Giovanni Muto, Isabella Sperduti, Steno Sentinelli, Manuela Costantini, Luca Pasquini, Michele Milella, Mustapha Haoui, Giuseppe Simone, Michele Gallucci, Ruggero De Maria, Désirée Bonci

**Affiliations:** 1 IRCCS, Regina Elena National Cancer Institute, Rome, Italy; 2 Department of Oncology and Molecular Medicine, Istituto Superiore di Sanità, Rome, Italy; 3 Department of Internal Medicine and Medical Specialties, “La Sapienza” University, Rome, Italy; 4 Institute of General Pathology, Fondazione Policlinico Universitario A. Gemelli e Università Cattolica, Rome, Italy; 5 Centre of core facilities, Istituto Superiore di Sanità, Rome, Italy; 6 Department of Urology, Humanitas University, Turin, Italy; 7 Genetic and Clinic Pathology Unit, University Campus Bio-Medico of Rome, Rome, Italy; 8 Department of Biosciences, Biotechnologies and Biopharmaceutics, University of Bari, Bari, Italy

**Keywords:** Renal cell carcinoma, Patient-derived xenografts, Reverse phase protein array; personalized therapy, Targeted therapy

## Abstract

**Background:**

Clear cell RCC (ccRCC) accounts for approximately 75% of the renal cancer cases. Surgery treatment seems to be the best efficacious approach for the majority of patients. However, a consistent fraction (30%) of cases progress after surgery with curative intent. It is currently largely debated the use of adjuvant therapy for high-risk patients and the clinical and molecular parameters for stratifying beneficiary categories. In addition, the treatment of advanced forms lacks reliable driver biomarkers for the appropriated therapeutic choice. Thus, renal cancer patient management urges predictive molecular indicators and models for therapy-decision making.

**Methods:**

Here, we developed and optimized new models and tools for ameliorating renal cancer patient management. We isolated from fresh tumor specimens heterogeneous multi-clonal populations showing epithelial and mesenchymal characteristics coupled to stem cell phenotype. These cells retained long lasting-tumor-propagating capacity provided a therapy monitoring approach in vitro and in vivo while being able to form parental tumors when orthotopically injected and serially transplanted in immunocompromised murine hosts.

**Results:**

In line with recent evidence of multiclonal cancer composition, we optimized in vitro cultures enriched of multiple tumor-propagating populations. Orthotopic xenograft masses recapitulated morphology, grading and malignancy of parental cancers. High-grade but not the low-grade neoplasias, resulted in efficient serial transplantation in mice. Engraftment capacity paralleled grading and recurrence frequency advocating for a prognostic value of our developed model system. Therefore, in search of novel molecular indicators for therapy decision-making, we used Reverse-Phase Protein Arrays (RPPA) to analyze a panel of total and phosphorylated proteins in the isolated populations. Tumor-propagating cells showed several deregulated kinase cascades associated with grading, including angiogenesis and m-TOR pathways.

**Conclusions:**

In the era of personalized therapy, the analysis of tumor propagating cells may help improve prediction of disease progression and therapy assignment. The possibility to test pharmacological response of ccRCC stem-like cells in vitro and in orthotopic models may help define a pharmacological profiling for future development of more effective therapies. Likewise, RPPA screening on patient-derived populations offers innovative approach for possible prediction of therapy response.

**Electronic supplementary material:**

The online version of this article (10.1186/s13046-018-0874-4) contains supplementary material, which is available to authorized users.

## Background

Renal Cell Carcinoma (RCC) is one of the mainly diagnosed cancers both in men and women (www.cancer.gov/types/kidney). According to the classification adopted by the World Health Organization (WHO, 2016) and by guidelines of the Union Internationale Contre le Cancer (UICC, 2017), RCC is classified in 16 subtypes. The three most common subtypes are i) clear cell RCC (ccRCC), accounting for approximately 75% of the cases, followed by ii) papillary RCC (pRCC), representing approximately 10% of the cases, and iii) chromophobe RCC (chRCC), which covers approximately 5% of all cases. The remaining subtypes are rare and represent less than 5% of the cases [1]. Surgery is the definitive treatment in the majority of cases, with an estimated survival rate of about 70% of clear cell RCC patients within five year post-surgery (UICC guidelines). Nonetheless, about 30% of localized tumors develop metastases within three to five years. Current adjuvant therapy protocols designed to prevent recurrence lack reliable prognostic indicators for determining patients who will benefit from the treatment. In addition, since many cancers are clinically silent, diagnosis is frequently made when the disease is already at an advanced stage. Unfortunately, the prognosis for locally advanced or metastatic RCC patients is generally poor [2]. Following surgery, the metastatic cases are treated with biological drugs and/or chemotherapy. In recent years, targeted therapy and immunotherapy have been demonstrated to be effective against advanced RCC [3, 4] (Guidelines from the European Association of Urology 2017). Selected tyrosine kinase inhibitors (TKIs) targeting angiogenesis and mTOR (mammalian target of rapamycin) pathways (i.e. Cabozantinib, Lenvatinib, Pazopanib, Temsirolimus, Everolimus, and antibody- mediated therapeutic compounds such as Bevacizumab), have significantly improved the survival of RCC patients [www.cancer.gov] [5–10]. However, the absence of reliable biomarkers of therapeutic efficacy, makes it difficult to predict which drug treatment will result in the highest benefit for patients and, to date, the treatment choice is mainly dependent on clinical evidences. A previous study has demonstrated, in pre-clinical settings, that high levels of mTOR pathway activation and plasmatic VEGF-A are associated with therapeutic efficacy [11–13]. However, these results were not paralleled by clinical evidence when the candidate antigen levels have been measured. Furthermore, a large portion of RCCs develop resistance to therapy. Mechanistically, drug failure could be a consequence of: i) compensatory feedback signaling loops following mTOR inhibition; ii) activation of alternative as well as synergistic pathways such as TGF-β after anti-angiogenesis therapy, and MAPKs/Ras signaling, respectively; iii) de novo somatic lesions, like mTORC-1 point mutations. Altogether, scientific and clinical evidences show that the recent use of diverse targeted drugs did not produce the envisaged curative efficacy, which is prevented by the gap of knowledge on the molecular mechanisms underlying RCC and by the lack of reliable and specific indicators of therapeutic response. Therefore, the discovery of predictive biomarkers and of new tools for monitoring therapy are imperatively needed in all phases of this neoplastic disease. To additionally complicate the scenario, most cancers are composed of a heterogeneous population of cells endowed with tumor-propagating capacity characterized by diverse, acquired genetic lesions, which warrant further investigation [12]. Stem-like sub-population of cells, responsible for distal spreading and therapy resistance, have been prospectively isolated from a variety of cancers and are used for in vitro disease modeling [14, 15]. Interestingly, this fraction of cells with stem-like properties has been shown to recapitulate the structures of original tissues in vitro by generating organotypic cultures as well as in vivo by forming tumor-like masses [16–18]. Tumor heterogeneity could be translated into diverse undifferentiated subpopulation, which may synergistically or independently concur to cancer aggressiveness, spreading and therapy resistance [19, 20]. Several evidences have implicated patient-derived xenograft (PDX) models in the definition of patient outcome and as drug testing tool in RCC [21–28]. However, rare evidences report renal cancer orthotopic PDXs generated from heterogeneous stem cell like enriched cells. Here, we show the isolation and propagation in vitro, of an undifferentiated heterogeneous population of RCC cells, able to generate masses, i.e. PDXs, when orthotopically injected into immunocompromised mice. In line with the embryonic origin of renal stem cells from the mesenchymal germ layer, the isolated cell populations showed undifferentiated, epithelial and mesenchymal markers when analyzed by flow cytometry. PDXs derived from high-grade tumors demonstrated multiple transplantation capacity after serial re-injection into immunocompromised mice. Differential activation of MAP-kinase and VEGF signaling pathways by Reverse-Phase Protein Array (RPPA) has been previously shown to be associated with tumor grade [29]. In our hands, the expression levels of several RPPA analytes in RCC stem-like cells, correlated with the probability of disease recurrence. Moreover, we found specific patterns of protein co-regulation suggestive of potential drug target candidates for innovative treatments. The innovative and efficient PDX system described here allows faithful propagation and phenocopy of RCC patient tumors, resembling parental morphology, grading and aggressiveness. The formation of PDXs per se correlates with poor patient prognosis thereby integrating a novel, reliable cell and tissue platform for translational research as well as a tool for disease monitoring. Our in vitro and in vivo models are likely to improve biomarker discovery and provide a useful approach for prediction of treatment efficacy and drug testing in RCC.

## Methods

### Samples collection and tumors dissociation

Tumor samples were obtained in accordance with consent procedures approved by ethical committees of Regina Elena National Cancer Institute in Rome. Case grading was defined and revised by our Pathologist according to ISUP 2013 and WHO 2016 guidelines.

Fresh tumor specimens were obtained immediately after surgery from all patients, according to approved protocols. All the data were analyzed anonymously throughout the study. Once the specimens arrived in the lab, each sample was divided into two parts: the first part was fixed in OCT and the second part was used to establish the PDX models. Other important clinical data, including clinic-pathological characteristics from each patient, were obtained from their medical archives and they are summarized in Additional file 1: Table S1A, B. Freshly used surgical specimens were washed several times with DPBS (Invitrogen Life Technologies Inc., Grand Island, NY) supplemented with metronidazole at 20% (Braun Melsungen, AG) and Antibiotic-Antimycotic at 4% (Sigma-Aldrich Inc., Saint Louis, MO). Tissue dissociation was carried out by mechanical dissociation of the tumor tissue with sterile scissor followed by an enzymatic digestion in Dulbecco’s Modified Eagle Medium High Glucose with L-Glutamine (Sigma-Aldrich Saint Louis, MO), supplemented with hyaluronidase IV (Sigma- Aldrich Saint Louis, MO) (2 μl/ml) and collagenase II (Invitrogen Life Technologies Inc., Grand Island NY) (10 μl/ml) for 45 min at 37 °C. Recovered cells were cultured in a serum-free medium containing 50 μg/ml insulin (Sigma- Aldrich Saint Louis, MO), 100 μg/ml apotransferrin (Sigma- Aldrich Saint Louis, MO), 10 μg/ml putrescine (Sigma-Aldrich Saint Louis, MO), 0.03 mM sodium selenite (Sigma-Aldrich Saint Louis, MO), 0.6% glucose (Sigma-Aldrich Saint Louis, MO), 5 mM HEPES (Sigma-Aldrich Saint Louis, MO), 0.1% sodium bicarbonate (Sigma-Aldrich Saint Louis, MO), 0.4% Bovine Albumin Cohn Fraction V (BSA) (MP Biomedical Santa Ana, CA), glutamine (Gibco-Invitrogen Life Technologies Inc., Grand Island, NY) and antibiotics, dissolved in DMEM-F12 medium (Gibco-Invitrogen Life Technologies Inc., Grand Island, NY) and supplemented with 20 μg/ml EGF and 10 μg/ml Bfgf (Peprotech catalog# AF-100-15 and catalog#100-18B). Non-treated flasks for tissue culture were used to reduce cell adherence and support growth as undifferentiated tumor spheres. The medium was replaced or supplemented with fresh growth factors twice a week, after few days cells started to grow forming floating aggregates. Cultures were expanded by mechanical dissociation of spheres, followed by re-plating of both single cells and residual small aggregates in complete fresh medium.

### Clonogenesis cultures

For colony forming assay, aggregates where gently dissociated with Tryple Express (Thermo Fisher), cultured or isolated by flowcytometer sorting, then embedded in Growth factor reduced (GFR) Matrigel (Corning) in presence of serum free stem cell basic medium supplemented with EGF and b-FGF factors or DMEM medium supplemented with FBS. 1000 cells/well for each conditions were used in triplicates.

### Cell lines

The human renal cancer cells 786–0 (CRL-1932) were obtained from the ATCC maintained in indicated medium at low passages and never kept in culture for more than six months. Caki-1 cells were kindly provided by Donatella Lucchetti (UCSC) and maintained in manufacturing indicated medium. Cell lines were cultured as monolayers at 37 °C and 5% CO2 .

### Flow cytometry and cell sorting

For flow cytometry analyses and cell sorting tumor spheres were dissociated as single cells, washed and incubated with the appropriate dilution of control or specific antibody. Antibodies used were V450 Mouse Anti-Human CD44 (561292), PE-Cy7 Mouse Anti-Human CD45 (557748), PE Mouse Anti- Human CD146 (550315), PE-Mouse Anti-Human CD24 (555428), FITC Mouse Anti-Human CD90 (561969), FITC-Mouse Anti-Human EpCAM (347197), APC-Mouse Anti-Human CD10 (332777), (all from BD Bioscience Bedford, Franklin Lakes, NJ) and Mouse monoclonal antibody [5D3] to Cytokeratin 8 + 18 (Abcam Cambridge,UK, 17139). After 45 min incubation, cells were washed. Analysis was performed using a FACS Canto flow cytometer (BD Biosciences). Cell Sorting was performed by FACS ARIA cytometer equipped with three lasers (488, 633, 407 nm) (BD Biosciences). All the cytofluorimetric acquisitions were analyzed by BD FACSDiva Software version 6.1.3 (BD Biosciences). For the sorting, cells were incubated with antibodies for 1 h then washed in PBS. Antibodies were used following manufacturing protocol indication for sorting experiment. TO-PRO3 (Thermo Fisher) dye was used for viability evaluation and used following manufacturing protocol indication. All the cytofluorimetric acquisitions were analyzed by BD FACSDiva Software version 6.1.3 (BD Biosciences).

### Lentiviral transduction

The lentiviral reporter vector TWEEN-Luc-GFP was constructed by using pRRL-CMV-PGK-GFP- WPRE (pTWEEN) as a backbone for subcloning a firefly luciferase NheI/XbaI cDNA fragment extracted from pGL3 (Promega) into the XbaI site at the 3′ of the CMV promoter [30, 31]. For the production of viral supernatant and cells infection we followed our internal optimized protocol [30, 31].

### RNA extraction and real-time qRT-PCR analysis

To investigate the mRNA levels of renal cancer stem cell related genes, total RNA was extracted using Trizol® total RNA isolation reagent (Gibco Life Technologies, Grand Island, NY) following the manufacturer’s protocol. One μg of RNA was reverse transcribed with M-MLV reverse transcriptase (Invitrogen Life Technologies Inc., Grand Island, NY) with random primers. cDNA was diluted 1:10 in the PCR reactions. *SOX2* expression levels were measured by quantitative real-time PCR using the SYBR Green assay (Applied Biosystems, Carlsbad, CA, USA) on a StepOne instrument (Applied Biosystems). *RRN18S* was used as reference endogenous gene (Applied Biosystems, Carlsbad, CA, USA).

*SOX-2* amplification was performed using the following primers:

*SOX2*- FW TACAGCATGTCCTACTCGCAG.

*SOX2* -RW GAGGAAGAGGTAACCACAGGG.

Values are expressed in terms of 2-ΔΔCT where ΔΔCT = ΔCTsample- ΔCTcalibrator.; ΔCT is the difference in threshold cycles between the mRNA and *RRN18S* amplicons, and CT is a parameter given by ABI PRISM 7700 Sequence Detector software by negative correlation with an internal reference (Applied Biosystem Life Technologies Inc., Grand Island, NY).

### Western blotting

Cellular pellets were lysed in RIPA buffer: 150 mM NaCl, 10 mM Tris-HCl, 1 mM EDTA and 1% Triton-X100 and protease inhibitors (Sigma-Aldrich Inc., Saint Louis, MO). Samples were resolved on 4–12% SDS-PAGE gels using a mini-gel apparatus (Bio-Rad Laboratories, Richmond, CA) and transferred to Hybond-C extra nitrocellulose (Amersham Pharmacia Biotech, Piscataway, NJ). Membrane was blocked for 1 h with 5% non-fat dry milk in TBS containing 0.05% Tween-20 and incubated over night with primary antibody. The primary antibodies used are: pAKT S473 (4058) and pERK T202/Y204 (9101) (all from Cell Signaling Technology, Danvers, MA) and GAPDH (G9545) (Sigma-Aldrich Inc., Saint Louis, MO). Washed filters were then incubated for 45 min with HRP-conjugated anti-rabbit or anti-mouse secondary antibodies (Amersham Pharmacia Biotech, Piscataway, NJ) and visualized by using an enhanced chemioluminescence detection system (ChemiDoc XRS+ (Bio-rad) with Image Lab software).

### Immunofluorescence assay

Renal cancer bulk cells were set down on slides by low speed cytospin. Typically, cells were fixed in 2% paraformaldehyde and permeabilized in 0.1% Triton X-100 (Bio-Rad Laboratories, Richmond, CA /) then incubated overnight at 4 °C with primary antibodies diluted in PBS containing 3% bovine serum albumin (BSA), 0.1% Triton X-100. After two washes in PBS, cells were incubated with Alexa Fluor-conjugated secondary antibodies for 30 min at room temperature in the dark, stained for 15 min with 4,6-diamidino-2-phenylindole (DAPI) (Invitrogen Life Technologies Inc., Grand Island NY), prepared in PBS 3% BSA, and mounted with Prolong-Gold antifade (Invitrogen Life Technologies Inc., Grand Island, NY). Slides were analyzed on a FV1000 confocal-microscope (Olympus, Tokyo, Japan, http://www.olympus-global.com).

### Immunofluorescence staining of FFPE

Clear cell renal carcinoma tissue analysis was performed as follows. Sections were deparaffinized, hydrated and then incubated in Citrate Buffer (SIGMA) for Antigen Retrieval at 96 °C water bath for 10 min. Tissues were washed in PBS and incubated with Glycine 1 M for 1 h. Sections were incubated with primary antibody anti-EpCAM (Dako Agilent) (1: 50) and anti CD146 (1: 50) (R&D) in PBS containing 3% BSA, 3% FBS and 0,1% Triton X-100 overnight at 4 °C. After washing in PBS, sections were incubated with a mouse alexa 555 and goat alexa 647-conjugated secondary antibody [1: 500 (Thermo Fisher) in PBS containing 3% BSA, 3% FBS and 0,1% Triton X-100] 1 h at 37 °C and then stained for 15 min with DAPI (Invitrogen) diluted in PBS 3% BSA, and subsequently mounted with Prolong-Gold antifade (Invitrogen). Slides were analyzed on a FV1000 Confocal microscope (Olympus, Tokyo, Japan) equipped with 40 × and 60 × oil immersion objectives by the Olympus Fluoview software.

### Immunohistochemistry (IHC) assay

Immunohistochemistry was performed on formalin-fixed paraffin-embedded or frozen tissues. Paraffin tissue samples derived from the xenografts were cut into 2-μm sections using a microtome LEICA SM 2000R (Advanced Research Systems Inc., Macungie, PA). Paraffin sections were dewaxed in xylene and rehydrated through a series of graded ethanol solutions and stained with Gill’s Haematolylin (Bio-optica, Milan) and Eosin (Bio-optica, Milan). The unmasking of bond sites was performed using a thermal bath at a temperature of 96 °C with a buffer specific for the primary antibody. The slides were incubated with the following primary antibody: PAX8 (Novus biologicals, Littleton, CO; NBP1–32440), Mouse Monoclonal [clone 56C6] (IgG1) to Human MME/CD10 (ScyTek Laboratories, Logan, UT), Monoclonal Mouse VIMENTIN Clone V9 (Dako, Santa Clara, CA; M0725) and Anti-Human Epithelial Membrane Antigen (EMA) (Novacastra Laboratories, Newcastel, UK; NCL-L-EMA), mTOR (Cell Signaling Technology, Danvers, MA; #2972) and VEGF Receptor 2 (Cell Signaling Technology, Danvers, MA; clone 55B11, #2479) antibodies. The reaction was performed using Bond Polymer Refine Detection kit (Leica, Milan, Italy) and DAB substrate chromogen (Dako Liquid DAB+ Substrate Chromogen System) (Dako, Santa Clara, CA) followed by counterstaning with Haematoxylin (Bio-optica, Milan). For IHC performed on frozen tissue the same protocol was followed skipping the dewaxing and unmasking steps.

### Animals

Six weeks old male of 20 g NOD Cg-*Prkdcscid Il2rgtm1Wjl*/SzJ (NSG) mice were purchased from Charles River Laboratories (Calco, LC, Italy) and were used for the injection of the clinical tumor samples depending on patient gender. They were housed in groups of four in isolated ventilated cages; food and water were provided ad libitum. All animal procedures were performed according to the protocol approved by the Health Institute of Sanity Animal Care Committee. Investigation has been conducted in accordance with the ethical standards and according to the Declaration of Helsinki and according to national and international guidelines and has been approved by the authors’ institutional review board.

### Establishment of a human renal cell carcinoma PDX model

For the sub-renal capsule (SRC) model, the animals for the injection were anesthetized with a mixture of ketamine (Ketavet Intervet Productions srl Aprilia Italy) (100 mg/kg) and xylazine (Rompum Bayer Leverkusen Germany) (10 mg/kg). Under sterile conditions, a skin incision of approximately 1 cm was made along the dorsal midline of an anesthetized mouse. With the mouse lying on its side, a body wall incision was then made slightly shorter than the long axis of the kidney. The left kidney was slipped out of the body by applying pressure on both sides of the organ using the forelimb and thumb. Injection of a mixture of renal carcinoma stem-like cells (1 × 105) from renal human carcinoma carrying Luciferase–EGFP gene reporters, resuspended in 20 μl of matrigel (BD Biosciences Bedford, MA) was administered with a 29-G needle in the subcapsular space of the kidney, taking care of not damaging the parenchyma. Phosphate-buffered saline was injected as surgery control. The kidney was then gently eased back into the peritoneal space; the body wall incision was closed using a 4/0 absorbable suture (Ethicon- Johnson &Johnson, Pomezia, Italy), while the skin incision was closed with surgical staples (Fine Science Tools, Heidelberg, Germany). Approximately 1 ml of saline solution was administered subcutaneously immediately after surgery. For in vivo imaging analysis, mice were injected intraperitoneally with 150 mg/kg D-luciferin (Caliper Life Sciences, Tremblay and France, France) 10 min before imaging and then they were anesthetized with a mixture of oxygen and isoflurane gas. A cryogenically cooled imaging system (IVIS 100 Imaging System, Xenogen Corporation Alameda, CA) was used for data acquisition. Whole animal imaging was used to monitor tumor growth; signal intensities were quantified as the sum of all detected photons. Mice with tumor intensities of 2.6 × 10^6^ were randomly assigned to two treatment groups (seven mice/group): [1] control, [2] sunitinib 40 mg/kg oral administration (gavage) 5 days a week for 3 weeks. Tumor growth was monitored every other day. At the end of the experiment, mice were sacrificed and tumors were embedded in optimal cutting temperature (OCT) medium and frozen at − 80 °C for histological analyses.

### Reverse-phase protein arrays (RPPA)

RPPA were constructed and analyzed as previously described [32]. Briefly, all cell lysates were printed twice in triplicate spots at concentrations of 0.5 μg/μl and 0.125 μg/μl on nitrocellulose- coated glass slides (GRACE Bio-Labs, Bend, OR) using an Aushon 2470 equipped with 185-μm pins (Aushon Biosystems, Billerica, MA), according to manufacturer’s instructions. Reference standard cell lysates were printed in 10-points dilution curves as procedural controls and as positive controls for antibody staining. A selected subset of the printed array slides were stained with Sypro Ruby Protein Blot Stain (Invitrogen, Carlsbad, CA) to estimate sample total protein concentration. After slide priming, immunostainings were carried out using a signal amplification kit (Dako, Santa Clara, CA) through an autostainer (Dako, Santa Clara, CA), according to manufacturer’s instructions. Arrays were probed with a library of more than 100 antibodies directed to total, cleaved and phosphorylated protein targets. Primary antibody binding was detected using a biotinylated goat anti-rabbit IgG H + L (1:7500) (Vector Laboratories, Burlingame, CA) or rabbit anti-mouse IgG (1:10) (DAKO) followed by streptavidin-conjugated IRDye680 fluorophore (LI-COR Biosciences, Lincoln, NE). Primary antibodies were validated for single band specificity by Western Blot using complex cellular lysates. Negative control slides were incubated with secondary antibody only. All Sypro and immunostained slides were scanned using a Tecan power scanner™ (Tecan Group Ltd., Switzerland). Acquired images were analyzed with MicroVigene v5.0 (VigeneTech, Carlisle, MA) for spot detection, local background subtraction, negative control subtraction, replicate averaging and total protein normalization. The software package JMP v7.0 (SAS Institute, Cary, NC) was used to carry out two-way unsupervised hierarchical clusterings with standardized data using Ward’s method. RPPA data were analyzed from TCGA GDC-database as indicated in the legends (https://tcga-data.nci.nih.gov/docs/publications/kirc_2013/).

### Statistical analysis

Descriptive data are presented as either means ± SD or median (range) for continuous variables, frequencies and percentages are reported for categorical variables. The receiver operating characteristic (ROC) curve analysis was used in order to find possible optimal cut-offs of different proteins capable of splitting patients into groups with different outcomes probabilities (progression or not progression). Performance characteristics (accuracy, sensitivity, specificity, areas under the curves -AUC) were evaluated by computing receiver operating characteristic (ROC) curves. Kaplan-Meier method was used to estimate survival curves and differences between subgroups was assessed by the log-rank test. Significance was defined at the *p* ≤ 0.05 level. The SPSS (version 21.0; SPSS Inc., Chicago IL, USA) was used for all the analyses. JMP v7.0 was used to perform Wilcoxon/Mann-Whitney non-parametric test for comparing quantitative variables. Graphpad Prism v5 was used to perform bar plots and 3 groups one-way analysis of variance. Significance was defined at the *p* ≤ 0.05 level. Concerning RPPA data, we compared samples from progressing versus non-progressing patients by receiver operating characteristic-ROC and Mann-Whitney test analyses.

## Results

### Establishing patient clear renal cell carcinoma in vitro cultures

**Fig. 1:**
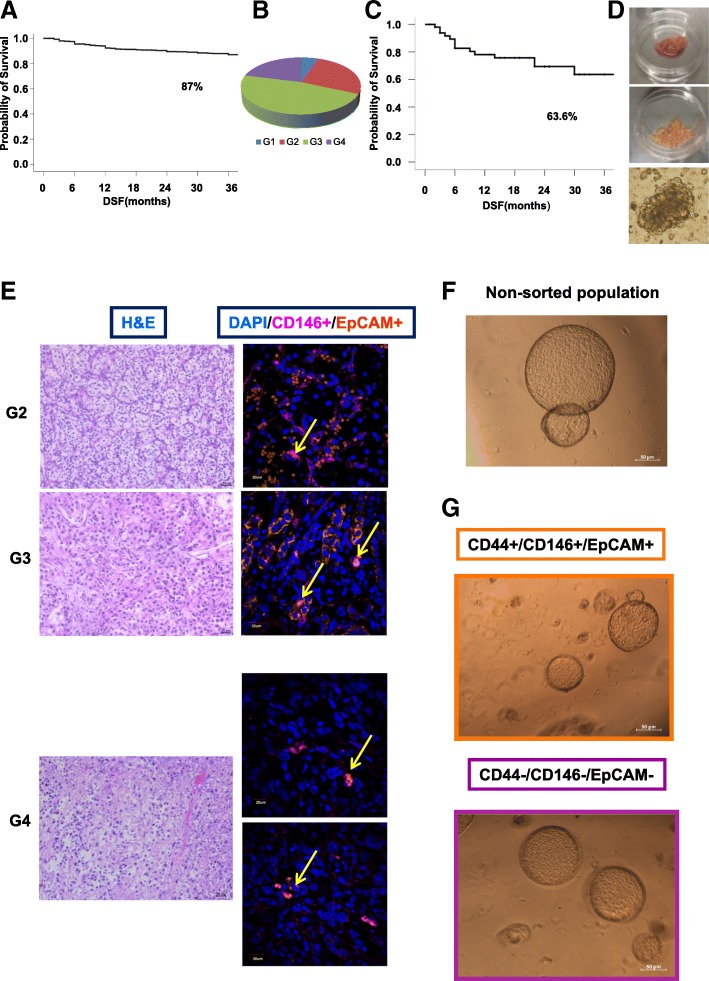
ccRCC cultures obtained from dissociation of fresh surgery patient specimens. **a** Kaplan-Meier analysis reporting disease free survival (DFS) of 1286 ccRCC patients (**b**) Pie charts showing the 57 collected fresh renal cancer surgery specimens. ccRCC were enrolled based on grade (G1, G2, G3, G4). **c** Kaplan-Meier analysis reporting DFS of 57 ccRCC patients enrolled for the study. **d** Images showing a fresh surgery tissue enzymatically dissociated and renal cancer spheroids structures. Microscope Magnification 10×. **e** Immunofluorescence analysis of EpCAM and CD146 antigens on clear cell renal cancer Formalin-Fixed Paraffin-Embedded (FFPE) tissues. Hematoxilyn and Eosin (H&E) and nuclear DAPI staining were used as controls. Three patients for each grading type were used and representative images were reported (G2,G3,G4). **f** Colony forming assay of fresh dissociated cancer tissues performed after one week of culture in serum-free stem cell-isolating medium supplemented with Epidermal Growth Factor (EGF) and basic Fibroblast Growth Factor (b-FGF). A representative image of colonies was reported. **g** Colony forming assay performed on cells after one week of culture in serum-free stem cell-isolating medium and purified as EpCAM+/CD146+/CD44+ and EpCAM-/CD146-/CD44- by sorting with FACS ARIA cytometer. A representative image of colonies of both sub-populations is reported. Orange (P6) and Pink (P5) boxes mirror sorting gating/plot density areas (Additional file 7: Figure S6A)

In RCC patients, TNM stage, tumour nuclear grade (G), and RCC subtype provide important prognostic information [UICC and International Agency for Research on cancer (IARC), ISUP 2013, WHO 2016 guidelines]. Clear cell RCC has a worse prognosis compared to the other subtypes and the five-year cancer-specific-survival (CSS) rate ranges from 91% down to 32% with increasing stage and histopathological grade. The CSS evaluated on a cohort of 1286 clear cell RCC cases (arrived at our Institute since 2001 up today) showed about 13% of recurrent cases within 36 months, and about 130 patients arrived with metastases at diagnosis (Fig. 1a and Additional file 2: Figure S1A, Panel 1). In order to define new prognostic markers and models for adjuvant or curative therapy testing, we decided to enroll a group of patients with poor prognosis and excluded all clear cell RCC cases with T1a from the study. The majority of these patients presented with favorable post-surgery prognosis. We collected fresh surgery specimens from 57 patients with clear cell tumors. The aforementioned cohort was selected so as to include all Grades (Additional file 1: Table S1A and Fig. 1b). Of the 57 cases, 6 cases arrived with metastases at diagnosis and 27% of cases recurred within 36 months (Additional file 2: Figure S1A, Panel 2). Due to our arbitrary selection, the enrolled group showed a reduced disease free survival time (Fig. 1c) when compared to the general collection of cases (Fig. 1a). Fresh specimens underwent enzymatic and mechanical disaggregation after 2–4 h from surgical resection and maintained in culture at low density in a serum-free, stem cell-isolating medium supplemented with Epidermal Growth Factor (EGF) and basic Fibroblast Growth Factor (b-FGF). Cells formed spheroid-like structures within 48 h (Fig. 1d). Such cellular aggregates were able to proliferate and duplicate once per week (data not shown). DAPI and 7-aminoactinomycin D staining showed that cellular spheroids were 70–80% viable and apparently devoid of apoptotic or suffering cells after one week of culture (Additional file 2: Figure S1B, C). Cytofluorimetric analysis of these cultures after one week of culture revealed a low-level contamination of hematopoietic cells and fibroblasts, as demonstrated by < 10% expression of both CD45 and CD90 markers (Additional file 2: Figure S1D). Angiogenesis is abundant in G2 and G3 while considerably less pronuonced in G4 renal cancers, as indicated by CD31 staining in patient tissues (Additional file 3: Figure S2A) and in RPPA-TCGA-tissue analysis performed on macro-dissected tumor tissues (Additional file 3: Figure S2B). Interestingly, in G4 cases epithelial cells express CD31 marker, suggesting a more undifferentiated phenotype (Additional file 3: Figure S2A, B). However, our in vitro culture did not showed angiogenesis marker expression such as CD31 and VE-Cadherin (VE-Cadh), while retaining a moderate and low levels of CD133 and CD105 stem cell markers, respectively (Additional file 3: Figure S2C). We further evaluated the expression of a panel of epithelial markers, including CK8–18 and CD10 (Additional file 2: Figure S1E), and we found elevated percentage of CD44 positive cells and concomitant expression of CD24 antigen in < 60% of the population (Additional file 1: Figure S1E). Notably, the cell spheroids also expressed EpCAM, an epithelial cell adhesion molecule associated with epithelial tumor phenotype and cancer spreading (Additional file 2**:** Figure S1e). Since advanced renal cancer oftentimes express mesenchymal markers, we extended the phenotypic characterization to CD146, a stem-cell marker, expressed by mesenchymal cells with marked plasticity and associated with cancer aggressiveness [33, 34]. In RCC-derived stem-like cell lines, CD146 levels are elevated a substantial fraction of the cell population and, since most of the cells are EpCAM positive, it is likely that both markers are co-expressed (Additional file 2: Figure S1E). These results suggest the presence of a highly undifferentiated sub-population retaining both epithelial and mesenchymal phenotype. The co-expression of epithelial and mesenchymal markers is in line with the well-documented acquisition of a sarcomatoid phenotype in advanced renal tumors [35]. We analyzed the fraction of cells with EpCAM and CD146 co-expression (EpCAM+/CD146+) along with tumor tissue grading. EpCAM marker seems to be more expressed in low-grade tumors, while CD146 is mostly express in G4 cases in parallel with the acquisition of a mesenchymal phenotype (Fig. 1e). EpCAM+/CD146+ cell percentage (ranging from 2 to 10% of total tissue population) seems increase with grading (Fig. 1e). RPPA-TCGA database analysis performed on macro-dissected tumor tissues revealed that the epithelial marker E-Cadherin decreases and Fibronectin expression increase in parallel with Grading (Additional file 4**:** Figure S3A). Furthermore, EpCAM+/CD146+ and CD44 marker were describe at mRNA level in different type of renal stem cells (Additional file 4: Figure S3B). Thus, we were able to isolate in vitro, from fresh surgery specimens, heterogeneous undifferentiated cancer cell populations likely responsible for tumor propagation. We further extended the analysis on commercial cancer lines, i.e. the 786–0 and Caki-1 cell lines derived from a primary tumor and a metastatic one respectively. Differently from stem-like cells, bulks commercial lines showed high CD24 expression coupled to a more differentiated phenotype. Moreover, we found that the levels of CD10 were elevated in the primary tumor line 786–0 but not in the metastatic Caki-1 cells. The remaining markers were comparable in both stem-like bulks and commercial cell lines (Additional file 2: Figure S1F). Interestingly, Caki-1 metastatic cells displayed low level of EpCAM and high CD146 expression, suggesting that epithelial-mesenchymal transition is likely associated with a metastatic phenotype (Additional file 2: Figure S1F). In order to compare our populations with standard in vitro cultivation methods, we selected 10 fresh cases (Additional file 1: Table S1B) that after enzymatic and mechanical dissociation tissues were separated in two conditions: i) serum-free stem cell-isolating medium supplemented with Epidermal Growth Factor (EGF) and basic Fibroblast Growth Factor (b-FGF); ii) DMEM (Dulbecco Modified Eagle Medium), Glutamine and FBS (Fetal Bovine Serum) supplemented medium. After three days of culture, both populations showed conspicuous cell membrane damage and death as detected by TOPRO3 staining (Additional file 4: Figure S3 C). Both types of culture retained EpCAM+/CD146+ and CD44 positive populations together with a hematopoietic cell population showing increased presence of granulocytic components in DMEM condition, as indicated by SSC-A (Side-scattered light) and FSC-A (Forward Scatter) (Additional file 5: Figure S4A). Cells maintained in the two conditions demonstrated different clonogenic capacity and a diverse type of colonies. In particular, DMEM cultures had a less clonogenic activity than cells maintained in stem serum-free medium, and produced a larger percentage of monolayer bidimensional colonies unable to form tridimensional spherical structures (Additional file 5: Figure S4B-D). In fact, monolayer structures (red boxes and Histograms; Additional file 5**:** Figure S4B, D) are generally associated to a more differentiated phenotype retaining inhibition contact property, while spheroids (Blue boxes and Histograms; Additional file 5: Figure S4B,D) are associated to highly undifferentiated tumor foci able to produce different sub-populations naturally belonged to diverse germ layers and able to reproduce all cancer components. The epithelial–mesenchymal transition (EMT) is a central driver of tumor malignancy and plasticity. Since EMT promotes the generation of stem-like cells, we decided to isolate cells retaining epithelial and mesechymal traits in both conditions.

EpCAM+/CD146+ and CD44 positive populations (EpCAM+/CD146+/CD44+) were purified in both culture conditions by FACS ARIA sorting and compared to triple negative relative counterparts (EpCAM-/CD146-/CD44-) (Additional file 6: Figure S5). Sorted cells maintained in DMEM-FBS condition were unable to form colonies, while EpCAM+/CD146+/CD44+ cells and triple negative in stem serum free medium showed clonogenic properties. In particular, EpCAM+/CD146+/CD44+ cells produced large spherical and bidimensional colonies able to invade and degrade the matrigel (Additional file 6: Figure S5). We completed the analysis one week after both culture conditions when the cell viability rose at about 80% (Additional file 4: Figure S3C). Both populations resulted free of hematopoietic contaminants and with similar SSC-A and FSC-A (Additional file 7: Figure S6A). The populations grown in stem serum-free medium retained expression gradient of EpCAM, CD146 and CD44 markers, together with an high proliferative and clonogenic rate (Fig. 1 F; Additional file 7: Figure S6A, B). DMEM/FBS populations showed high levels of all antigens while reduced their growth and clonogenic capacity (Additional file 7: Figure S6A, B). This may be in line with differentiation stimuli promoted by FBS. Also in this case, we isolated EpCAM+/CD146+/CD44+ cells selecting high expression levels in both culture conditions by cytometer analysis. Both sorted EpCAM+/CD146+/CD44+ and triple negative cells grown in serum-free medium were able to generate spheroidal colonies (Fig. 1f, g; Additional file 7: Figure S6B, C). However, the two populations showed a considerably different clonogenic capacity, both in terms of colony number and size (Additional file 7: Figure S6C, D). This is in line with the proposed stem-like potential of our bulks, which appears composed by multi-clonal and heterogeneous populations with enhanced proliferative and clonogenic properties.

### Reverse phase protein Array (RPPA) relative quantification

To further characterize our cells, we analyzed stem-like cell populations isolated from 20 different patients (Additional file 8: Table S2) by means of Reverse Phase Protein Array (RPPA) and performed relative quantification of 123 (phosho-)proteins (Additional file 9: Table S3) representative of most cancer-related pathways. The majority of antigens analyzed by RPPA displayed detectable (blue) expression levels while only a minor part were not measurable (black, Additional file 9: Table S3). In details, RCC-derived cells featured activation of Receptor Tyrosine Kinases (RTKs) associated with stem and embryonic cell markers (Additional file 10: Figure S7A). Of note, G4 samples demonstrated the highest levels of the embryonic marker SOX2. This is in line with the acquisition of an undifferentiated state and aggressive phenotype of the G4 tumors. Increased expression of SOX2 was confirmed in high grades by Real-Time PCR and immunofluorescence (Additional file 10: Figure S7B, C).

**Fig. 2:**
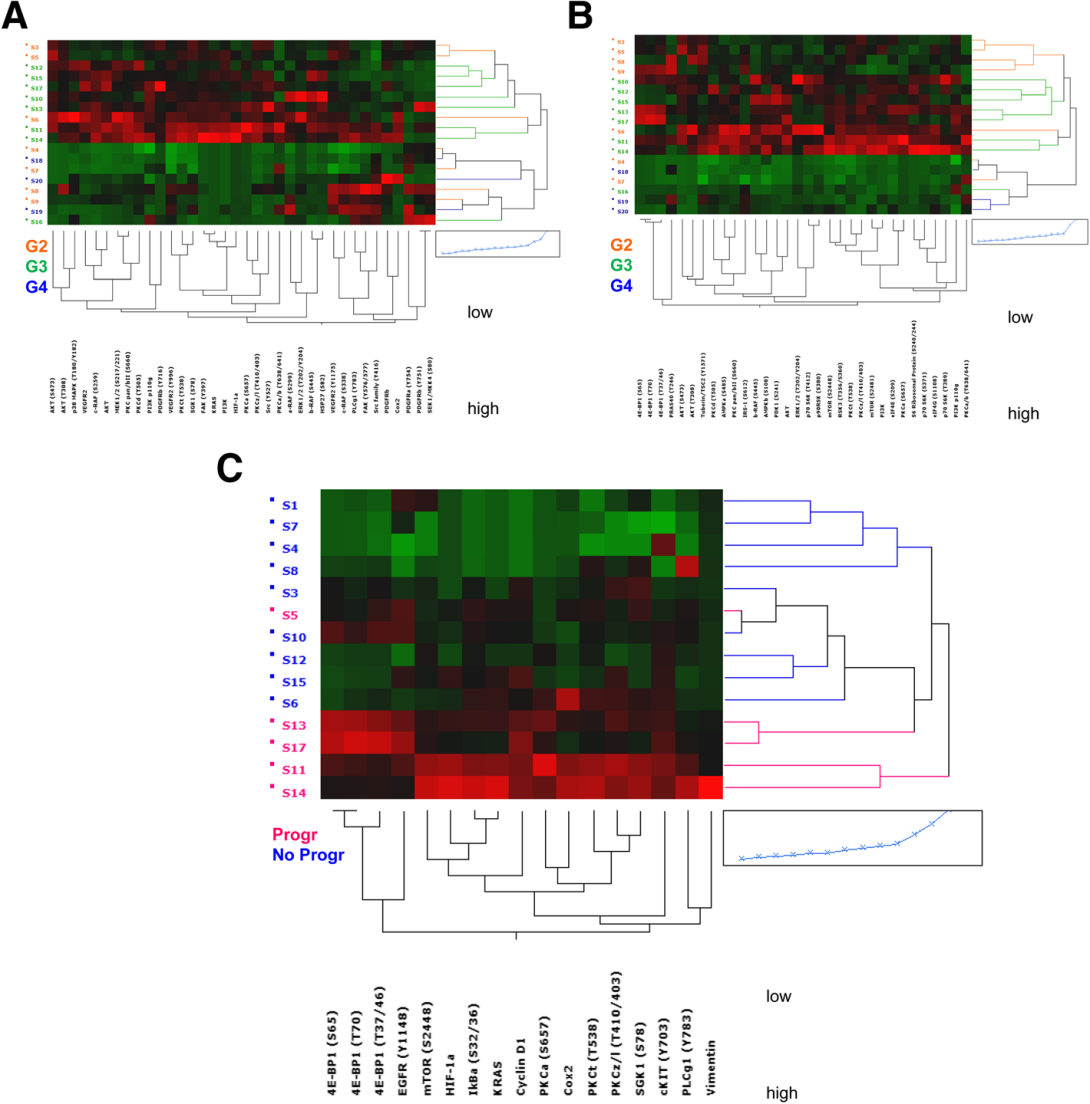
Reverse Phase Protein Array (RPPA) relative quantification. **a** Two-way unsupervised hierarchical clustering of 18 samples classified by grading as G2 (*n* = 7), G3 (*n* = 8) and G4 (*n* = 3) for the expression of endpoints belonging to the angiogenesis pathway showed as heatmap. **b** Two- way unsupervised hierarchical clustering of 18 samples classified by grading as G2 (n = 7), G3 (n = 8) and G4 (n = 3) for the expression of endpoints belonging to the mTOR pathway showed as heatmap. **c** Two-way hierarchical clustering of 14 samples classified as progressive (Progr. *n* = 5) and non-progressive (No Progr. *n* = 9) tumors and with a documented follow-up within 24 months clustered for the expression of 17 significant endpoints after non parametric test. Significant *p* value ≤0.05. In all the heatmaps the expression levels are represented as red (high) or green (low), with average values in black

The use of drugs targeting angiogenesis as well as mTOR inhibitors as post-surgery adjuvant treatment is largely debated [36]. Therefore, we included in our RPPA panel antigens related to these two signaling cascades, and analyzed their association with tumor grade. G3 but not G2 and G4 tumors, showed a marked increase of total and phosphorylated proteins involved in both mTOR and angiogenesis pathways (Fig. 2a, b). Conversely, G3 tumors demonstrated elevated though heterogeneous expression levels that warrant further investigation in terms of a potentially efficacious therapeutic approach. Mechanistically, the differential expression of RTKs and embryonic stem cell markers in the cases analyzed herein may account for the frequent failure of anti-angiogenesis and mTOR-targeted drugs in advanced renal tumors and may underlie the development of therapeutic resistance. Notably, since our RPPA data were obtained on RCC-derived stem-like cells, we sought to confirm the results on commercial RCC cell lines and RCC tissue specimens. To this end we analyzed, on G3 and G4 samples, the levels of selected, key (phospho-)proteins by Western Blotting or immunohistochemistry (Additional file 11: Figure S8A, B). We further analyzed sorted EpCAM+/CD146+/CD44+ and triple negative cells grown one week in serum-free medium as compared with non-sorted population maintained in both types of medium. Western blotting analysis demonstrated the upregulation in DMEM/FBS condition of several (phospho-)proteins, in line with the putative more differentiated phenotype (Additional file 11: Figure S8C).

In order to find RPPA profiles predictive of tumor recurrence, we focused our analyses to a subset of patients with documented 24-months follow-up after surgery. Among these patients, 5 showed evidence of progression (Recurrent) and 9 remained disease-free after surgery (non-recurrent). Interestingly and despite the poor sample size, we found a set of (phospho-)proteins allowing almost-clear-cut stratification of recurrent versus non-recurrent cases (Fig. 2). Table 1 reports the list of analyzed RPPA endpoints showing significantly different levels when comparing recurrent and non-recurrent cases. Therefore, such subset of (phospho-)proteins is ultimately associated with RCC neo-formations and may include promising candidates for prediction RCC recurrence. The aforementioned list of likely biomarkers and our present approach may ultimately guide the application of adjuvant therapy in patients at high risk of progression [25]. Of note, HIF-1 alpha is reportedly associated with cancer progression and with up-regulation of several genes able to promote angiogenesis [37, 38], including VEGF/VEGFR, which is among the pathways currently selected for targeted therapy. Interestingly, recurrent patients retained activation of angiogenesis-related signaling, which supports the idea that agents blocking this pathway may be effective in these patients (Additional file 11: Figure S8D). The levels of HIF-1 alpha and phospho-mTOR (S2448) antigens alone allowed efficient discrimination of recurrent and non-recurrent patients (Fig. 2c) with a high predictive power, as shown by the elevated Area Under the Curve (AUC) value after Receiver Operating Characteristic (ROC) curve analysis (Additional file 11: Figure S8E). Although, dedicated clinical trials should be conducted for assessing robustness of correlation between biomarker expressions, poor prognosis and therapeutic response These data support the translation of our approach into the clinics so as to i) molecularly characterize RCC patients, ii) rationally guide the post-surgery adjuvant therapy and iii) pave the way towards improved clinical trial designs [39, 40].

**Table 1:** List of significantly different endpoints after Mann-Whitney statistical analysis of non- progression (No Progr) versus progression (Progr) subgroups, together with their relative *p* values (significant *p* < 0.05)

SIGNIFICANT PROTEINS Progr vs No Progr	*p* value
Vimentin	0.004
HIF-1α	0.005
4E-BP1 (T70)	0.006
Cyclin D1	0.006
4E-BP1 (S65)	0.009
4E-BP1 (T37/46)	0.009
EGFR (Y1148)	0.014
KRAS	0.018
PKCθ (T538)	0.023
IkBα (S32/36)	0.027
mTOR (S2448)	0.028
PLCγ1 (Y783)	0.028
PKCα (S657)	0.028
PKCζ/λ (T410/403)	0.028
SGK1 (S78)	0.028
Cox2	0.028
cKIT (Y703)	0.039

### Patient-derived renal cancer xenografts (PDX) recapitulate parental tumors

**Fig. 3:**
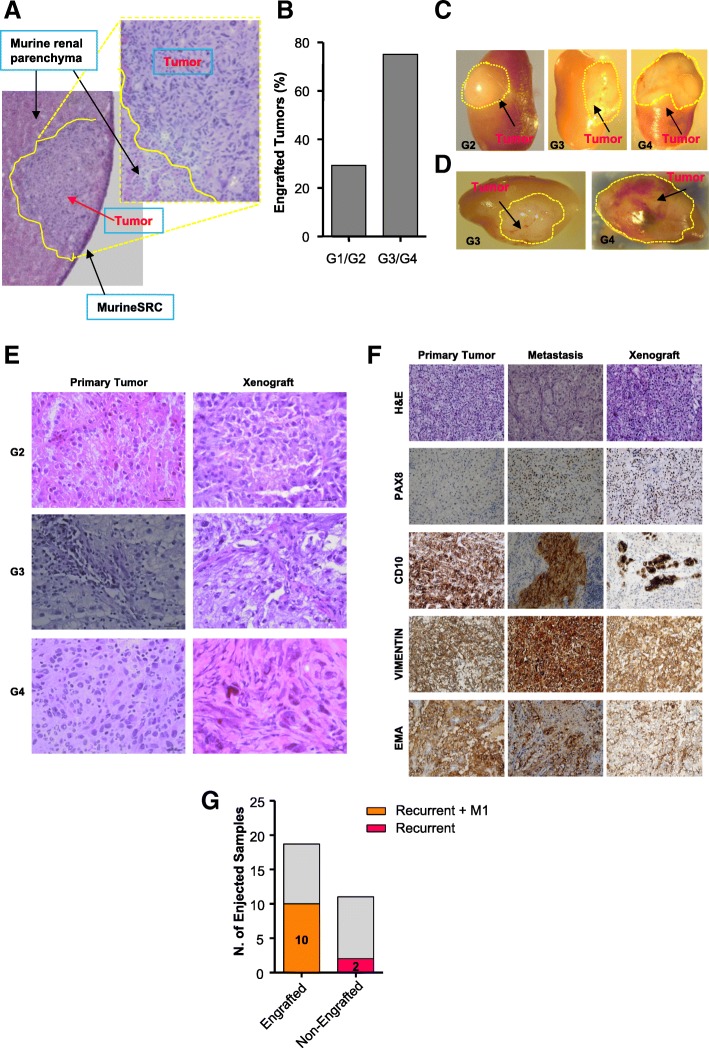
PDX model establishment from ccRCC injection. **a** Hematoxylin and Eosin staining of human tumor engrafted in murine models. A representative image of kidney PDX was reported. Microscope Nikon Eclipse E1000 10X and 20X (**b**) Graph reporting the percentage of patients who engrafted when orthotopically injected in mice and distributed following grading (3 G1; 7 G2; 13 G3; 7 G4). Eight mice for each patient were injected and all 18 tumors on 30 which were evaluated as engrafted developed tumor masses on ≥ of 6 mice. Tumors declared unable to engraft did not produce, at all, tumor masses. **c** Representative images (1 × 0.63) of the PDXs excised 90 days after injection by Stereomicroscope (Olympus SZX10,XCX50). One representative image for G2, G3 and G4 types was reported. **d** Representative images (1 × 0.63 and 1.25) of aberrant neo-angiogenesis formation in PDXs by Stereomicroscope (Olympus SZX10, XCX50 camera). **e** Hematoxylin and Eosin staining of PDXs versus parental primary patients. One representative tumor for each grade (G2, G3, G4) was reported. The staining was executed on OCT frozen samples. **f** Hematoxylin and Eosin and anti-PAX8, CD10, Vimentin and EMA staining were reported on formalin-fixed and paraffin- embedded parental primary, metastatic tissues and PDXs. Primary, metastatic and PDX tissues were obtained and shown from one representative patient. Microscope Nikon Eclipse 55i, magnification 20×. **g** Histogram showing the number of engrafting tumor populations evaluated over 30 injected patient samples and correlated with patient recurrence frequency calculated as development of metastases after surgery. Orange color represents recurrent (n = 7) and metastatic (n = 3) patients in the engrafted group (*n* = 18). Pink color represents recurrent patients (n = 2) in the non-engrafted group (*n* = 12) for a total of 30 injected samples

Following one week of in vitro culture, thirty cases (Additional file 12: Table S4) were orthotopically injected into NOD.Cg-Prkdc scid Il2rg tm1Wjl /SzJ (NSG) mice. The cultures showed a viability ≥70% (as detected by 7-aminoactinomycin D staining) at the moment of murine inoculation. The injected cells were able to home into and invade the host parenchyma, forming patient-derived chimeric masses (PDXs) (Fig. 3a). After three months following injection, mice were sacrificed, cancer masses explanted and analyzed. Isolated cancer populations engrafted with high efficiency (18/30=60%) in murine kidney. G3/G4 tumors demonstrated higher engrafting capacity (75%) and increased mass sizes when compared to low grade G1/G2 cases (Fig. 3b, c). Xenografts paralleled tumor aggressiveness through i) increased proliferation and ii) augmented aberrant malformed de novo vascularization at cancer periphery which was particularly evident in G4 tumors (Fig. 3c, d). PDXs exhibited morphological and grading features strictly resembling parental human tumors, as shown by Hematoxylin and Eosin staining of both primary patient specimens and the corresponding transplanted murine masses (Fig. 3e; Additional file 13: Figure S9). In order to characterize our collection of PDXs, we evaluated the expression of several markers with diagnostic relevance, such as PAX8 (Paired Box G8), Vimentin, CD10 and EMA (Epithelial Membrane Antigen). Expression of Vimentin and EMA in PDXs matched the levels found in parental primary tumors. Conversely, CD10 and PAX8 displayed lower and higher levels, respectively, compared to those found in their parental tumors and the expression of both antigens showed high similarity with parental metastatic tissue, in line with the idea that bulk populations were enriched for metastatic and aggressive cells (Fig. 3f; Additional file 13: Figure S9) [41, 42]. Out of the 30 injected bulk cell populations, 9 originally derived from patients that relapsed after surgery treatment and 3 were M1 *at esordium*. Interestingly, 7 of these recurrent cases together with 3 M1 patients, significantly contributed to the fraction of samples (*n* = 18) with tumor-forming ability, while only 2 recurrent cases were part of the non-engrafting group of tumors (*n* = 12) (Fig. 3g). Disease-free survival time of 30 injected cases showed a trend similar to their engraftment capacity in mice (Additional file 14: Figure S10A). Thus, the probability of homing into mice may be associated with poor prognosis and recurrence. Of note, three cases demonstrated a metastatic phenotype three months after the first injection (F1). In details, a G2 type at diagnosis showed a size comparable to a G3 tumor when injected in mice and a G3 type developed metastases in the murine hosts, similarly to its parental patient tumor, which underwent relapse after 24 months (data not shown). A similar scenario was found for a G4, M1, ccRCC case (as diagnosed before surgery), which was paralleled by a metastatic disease after engraftment in the murine host. Altogether, our results strongly support the exploitation of PDX model systems to predict the outcome of RCC patients and, in particular, tumor recurrence.

### Long lasting transplant of PDXs

**Fig. 4:**
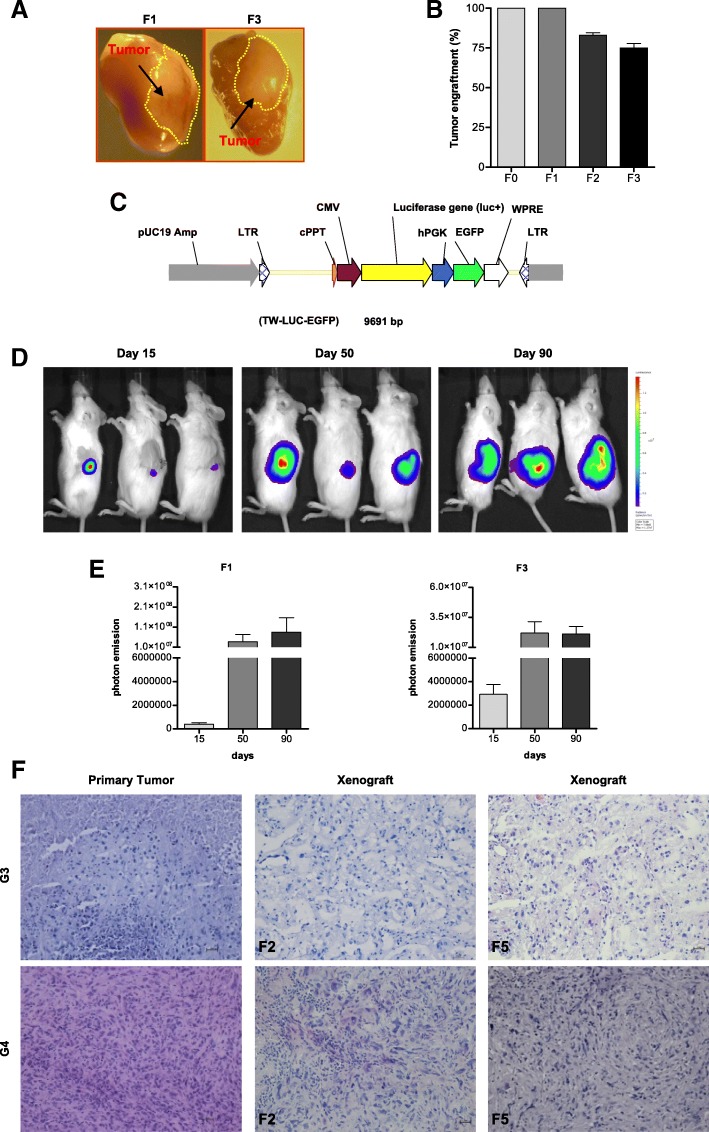
PDX serial transplants. **a** Representative images of PDXs serially transplanted. F1 and F3 transplant generations were evaluated 6 and 12 months after first injection in mice, respectively. **b** Histogram showing the percentage of engraftment from F0 until F3 for three G3 and three G4 tumors when orthotopically injected in NSG mice. Ten mice for each tumor (three G3 and three G4) have been injected for each transplant. In F0 and F1 all tumors engrafted in all mice. In F2 and in F3 all tumors (same situation in G3 and G4 tumors) engrafted but the percentage represents number of mice which generated tumor xenograft-masses calculated over total injected. **c** Schematic representation of the lentiviral vector TW-LUC-EGFP. **d** Representative images of Luciferase expression of ccRCC population (F0) injected mice acquired by IVIS imaging system at 15, 50, and 90 days after first injection. **e** Luciferase Photon emission analysis of xenograft tumors at 15, 50, 90 day during F1 and F3 transplant generations, respectively, by IVIS imaging system. For the experiments five mice per G3 and G4 tumors were used. Data were reported as mean and Standard Deviation (**f**) Representative Haematoxylin and Eosin staining of two PDXs at F2 and F5 transplant generations were reported. F5 represent 18 months after first injection. Microscope Nikon Eclipse 55i, magnification 20×. Each F represents murine recipient generation of 3 months (90 days). F0 represents 3 months after first injection; F1 represents 3 months after second injection; F2 represents 3 months after third injection; F3 represents 3 months after fourth injection and so on

It is acknowledged that a sub-population of cancer propagating-cells is ultimately responsible for distal tumor spreading and therapeutic resistance [43]. In order to demonstrate that our RCC-derived cultures are enriched for aggressive tumor-propagating cell populations, we performed in vivo serial transplantation of the pre-engrafted masses. To this end, RCC-derived cell culture samples obtained from patients with different grading (*n* = 3 for G1, G2, G3 and G4 cases) were orthotopically injected in vivo. In order to avoid clonal selection, PDXs generated from the same patient were explanted, subjected to mechanical and enzymatic dissociation and pooled together for re-injection into renal capsule of new murine hosts after maximum 48 h of in vitro culture. Only G3 and G4 cancer types produced tumors following reinjection into new murine recipients (data not shown). In addition, to determine the maximum number of sustainable serial transplants into new murine generations, 3 G3 and 3 G4 tumors were injected into mice (F0). The injected bulk populations retained capacity to form neoplastic masses until at least the F3 murine generation, i.e. 9 months after the first injection (Fig. 4a, b). These data demonstrated that tumor bulks were enriched for cells endowed with long-term tumor-propagating ability and suggest that a high tumor grade is associated with an increased aggressive behavior of RCC cells. In order to better estimate the capability of our system to propagate patient material, 3 bulk populations obtained from two G3 and one G4 tumors were transduced with a lentiviral vector (TW-LUC-EGFP) (Fig. 4c) containing both Enhanced Green Fluorescent Protein (EGFP) and the Luciferase gene for cytofluorimetic analysis and in vivo imaging, respectively. Infected cells (80–90% cells were EGFP positive after infection, data not shown) were orthotopically injected in mice, and F1-generation tumors were serially transplanted into recipient mice (Fig. 4d). Transplanted tumors were able to home and propagate efficiently in mice and displayed exponential growth as assessed by monitoring the localized production of luminescent signal using an in vivo imaging system (IVIS) (Fig. 4d, e). Notably, two bulk populations showed an increased tumor-propagating capacity along multiple rounds of transplantation, up to F6 generations, corresponding to a total period of 18 months. During such a period of serial transplantation, the tumors preserved their original phenotypical features, i.e. tumor grade and histology, as demonstrated by Hematoxylin and Eosin staining (Fig. 4f).

Taken together, these data underpin the opportunity to dispose of a reliable source of patient material for over 9 months and exploit such sample material for molecular analyses as well as in vitro and in vivo drug testing. Our model system may recapitulate the original patient and exploited as a platform for drug testing able to predict therapeutic responses and prevent unnecessary treatments.

In order to understand whether RCC-derived PDXs could be used as a model platform for drug testing, we dissociated and maintained in culture a fresh specimen of G4 sarcomatoid tumor, explanted from a patient with metastases *at esordium* (pT4, N1, M1, abdominal LN, liver, bone). In line with sarcomatoid phenotype, the bulk cell population showed elevated expression of CD44, coupled to moderate levels of CD146, EpCAM, CD24 and CD90 (Additional file 14: Figure S10B). Following one week of in vitro culture, cells were transduced with TW-LUC-EGFP vector and orthotopically injected into the renal capsule of NSG mice. The infected cell population was able to engraft with high frequency and generated tumor masses after 3 months. Such formed tumors were explanted, dissociated, pooled and re-injected into new murine propagating generations until F4 (i.e. 12 months post-isolation). The patient from whom this specimen was isolated underwent cytoreductive nephrectomy followed by Sunitinib (standard dose), but experienced rapid disease progression (bone, peritoneum) after treatment. To mirror patient treatment, chimeric-mice obtained with serial transplants of the parental tumor were treated with either drug-vehicle or Sunitinib for 21 days. In details, mice were orally administered by gavage, 5 days a week for 3 weeks, with either placebo or Sunitinib at a dosage of 40 mg/Kg. The minimum tumor burden required to start treatment was calculated as per photon counts in the range of 2–4 × 10^6^ obtained by the IVIS system. Treated and control mice were evaluated after three weeks from initial treatment by IVIS system (Additional file 14: Figure S10C). A significant increase in the amount of signal generated by accumulation of photons, showed that tumor growth was comparable in the control- and Sunitinib-treated group (Additional file 14: Figure S10D). The lack of efficacy after Sunitinib treatment in our PDX model system mirrored the therapeutic resistance displayed by the patient. Further analysis of cellular morphology by Hematoxylin and Eosin staining performed on explanted tumor masses, confirmed the resistance to Sunitinib therapy (Additional file 14 :Figure S10E). Altogether, these data suggest that PDX models may allow accurate prediction of patients’ therapeutic responsiveness. Additionally PDXs represent i) a useful tool to avoid the side effects of unnecessary therapeutics and ii) an experimental platform to test new compounds in the clinical trial settings. Finally and most important, the ability to map a wide variety of intracellular pathways by RPPA and identify specific drug targets in patient bulk cell populations (Additional file 14: Figure S10F) enable us with broad exploration of novel pharmacological approaches.

## Discussion

Renal cell cancer represents 2–3% of all cancers in Europe and during the last two decades its incidence significantly increased [44, 45]. Curative surgery is still the most efficacious treatment strategy. However, prognosis worsens with stage and histopathological grade and about 30% of clear cell RCC patients relapse within three to 5 years from surgery. To date, there are no adjuvant treatments available for reducing tumor recurrence and the opportunity to use angiogenesis inhibitors as post-surgery preventive treatment is debated [36]. Advanced RCC forms are primarily treated with mTOR inhibitors, anti-angiogenesis drugs and chemotherapy. Molecular indicators for therapy assignment are lacking and, unfortunately, several patients are either not responsive or acquire resistance. Tumor genomic heterogeneity is one of the main causes of therapeutic failure. In the era of innovative technologies and intelligent, targeted drugs, personalized therapy is at the horizon. In this scenario, therapy should perfectly fit with the intra-tumor diversity of an individual patient. It is of critical importance to find novel reliable and faithful models for the definition of individualized therapeutic protocols and the discovery of bioindicators for cancer monitoring. In this present work, we established a new in vitro type of culture from freshly dissociated RCC specimens, grown in a serum-free medium designed for stem-cell isolation. Much evidence shows that intratumor heterogeneity and tumor-protective microenvironment are key challenges in cancer medicine [19, 20]. In order to preserve the most aggressive and representative cancer populations, we avoid to isolate sub-populations expanding in vitro and in vivo patient bulks. Our heterogeneous multiclonal cells were enriched in stem-like and undifferentiated cells as demonstrated by cytoflurimetric and RPPA analyses. They may represent new renal cancer models based on the enrichment of all cells responsible of tumor aggressiveness. We found activated mTOR and angiogenesis pathways in G3 tumors, while aggressive G4 forms showed reduced levels of activation of these signaling cascades. This may be in line with the diminished sensitivity of the sarcomatoid phenotype to mTOR and angiogenesis inhibitors. Next, we established an orthotopic patient-derived cancer xenograft model by injecting these heterogeneous populations under the renal capsules of NSG mice. The so-formed PDXs robustly recapitulated parental cancer grading, size and aggressiveness, while showing a phenotype similar to metastatic tissues, as demonstrated by immunohistochemical analysis. Thus, isolated bulks are likely enriched for undifferentiated and aggressive cellular subpopulations, ultimately responsible for tumor spreading and therapeutic resistance. We performed multiple rounds of PDX transplants demonstrating that these bulks contain a significant proportion of tumor-initiating cells. Moreover, here we show that such PDX can be serially transplanted in vivo for a period > 18 months and used for drug testing of paralleled treatment cycles in advanced stage patients. These results suggest the potential application of a new tool for therapeutic testing ahead of patient treatment, thus preventing the toxicity of inefficacious treatments. In order to generate a panel of candidate therapeutic targets, we exploited the considerable throughput of RPPA, which allowed the analysis of total and phosphorylated proteins selected among key players in cancer-related pathways and among targets of conventional and innovative therapeutic treatments.

Our approach represents a pioneering method to explore the activation status of a broad spectrum of signaling cascades and eventually discover targetable cancer cell dependencies. Indeed, we demonstrate the generation of valuable experimental model systems as well as the long-term preservation of patient-derived tissues for use as preventive therapeutic testing. In addition, by exploiting a sophisticated (phospho-)proteomic technology for simultaneous screening of hundreds of analytes, we discovered novel therapeutic targets as well as potential biomarkers of RCC recurrence. Therefore, we believe that our data will significantly contribute to the design of novel, informative clinical trials and, by providing a rationale for the definition of personalized therapeutic strategies, will improve our knowledge on renal cancer.

## Conclusions

### The promise of personalized medicine—Targeting treatments based on patients

The promise of precision medicine is that treatments in a not too far future will be customized for each patient. Many efforts are currently dedicating to create new tests that will help to decide the most appropriated treatment reducing unnecessary drug side effects and national public cost of the health. In this contest the analysis of tumor propagating cells may help improve prediction of disease progression and therapy assignment. The possibility to test pharmacological response of ccRCC stem-like cells in vitro and in orthotopic models may help to create new monitoring approaches and to define pharmacological profiling for future development of more effective therapies.

## Additional files

Additional file 1: **Table S1.** (A) Clinical features of 57 collected ccRCCs patients including: 3 G1; 15 G2; 27 G3 and 12 G4. B. Clinical features of 10 collected ccRCCs patients used for sorting experiments. (ZIP 880 kb)

Additional file 2: **Figure S1.** (A) (PANEL1) Table of 1286 ccRCC patient distribution: 1013 tumor free patients at 36 months from surgery, 130 metastatic (M1) patients at diagnosis time and 143 recurrent patients at 36 months after surgery were reported. (PANEL2) Table of 57 ccRCC cancer patient distribution: 37 tumor free patients at 36 months from surgery, 6 metastatic (M1) patients at diagnosis time and 14 recurrent patients at 36 months from surgery were reported. (B) Representative immunofluorescence of DAPI-stained tumor derived spheroids. (C) Representative image of 7- aminoactinomycin D staining (7AAD) of in vitro isolated populations by flow cytometry. (D) Table reporting distribution of specific antigen expression percentages (%) in all studied ccRCC populations. (E) Representative images of flow cytometry analysis showing the expression of the epithelial and undifferentiated cell markers EpCAM, CD24, CD10, CD90, CD44 and CD146 mesenchymal stem cell markers in ccRCC isolated populations. Background staining was calculated by using appropriate isotype controls. (F) Flow cytometry analysis of cell lines 786–0 and Caki-1 representative of primary and metastatic tumor, respectively. One representative staining of three independent experiments is shown. (PDF 243 kb)

Additional file 3: **Figure S2.** (A) Hematoxylin and Eosin (H&E) and CD31 staining of formalin-fixed and paraffin- embedded (FPPE) of primary tumors. Three patients for each grading were analyzed. A representative image for samples is reported. (B) RPPA-TCGA elaboration of CD31 expression. Data were obtained from macrodissected clear cell renal cancer tissues (GDC-database-https://tcga-data.nci.nih.gov/docs/publications/kirc_2013/) and reported for grading, stage and for progression rate by RPPA. (C) Representative images of flow cytometry analysis showing the expression of the endothelial CD31, VE-Cadherin (VE-Cadh) and putative stem cell markers (CD133, CD105) in ccRCC isolated populations. The analysis was combined with CD44 expression. Background staining was calculated by using appropriate isotype controls. (PDF 402 kb)

Additional file 4: **Figure S3** (A) RPPA-TCGA elaboration of E-Cadherin and Fibronectin expressions. Data were obtained from macrodissected clear cell renal cancer tissues (GDC-database-https://tcga-data.nci.nih.gov/docs/publications/kirc_2013/) and reported for grading, stage and for progression rate by RPPA. (B) mRNA level elaboration of EpCAM, CD146(MCAM) and CD44 antigens. Data were obtained from GSE48550 microarray and were analyzed on different kinds of renal stem cells. (C) TOPRO3 staining for cell viability evaluation of populations maintained for three days (upper panels) and one week (Lower panels) in serum-free stem cell-isolating medium supplemented with Epidermal Growth Factor (EGF), basic Fibroblast Growth Factor (b-FGF), DMEM (Dulbecco Modified Eagle Medium), Glutamine and FBS (Fetal Bovine Serum) supplemented medium and evaluated by cytofluorimetric analysis. Blue and Black areas represent vital and dead cells respectively. (PDF 389 kb)

Additional file 5: **Figure S4.** (A) Fresh dissociated tissues maintained for three days in serum-free stem cell-isolating medium supplemented with Epidermal Growth Factor (EGF), basic Fibroblast Growth Factor (b-FGF), DMEM (Dulbecco Modified Eagle Medium), or in Glutamine and FBS (Fetal Bovine Serum) supplemented medium, and analyzed by cytofluorimetric analysis. CD45 (PE-Cy7), CD146(PE), CD44 (H450-Pacific Blue) and EpCAM(FITC) antigens were analyzed. TOPRO3 was used for gating vital cells. (B-C) Images and clonogenic population percentage of cells maintained in both conditions after three days of culture by Colony forming assay. Colonies distinguished on the basis of their shape in the two conditions: spheroidal (blue box) and bidimensional (red box). (D) Percentage of colonies distinguished on the basis of their shape in the two conditions was reported: spheroidal (blue box) and bidimentional (red box) such as in B. (PDF 229 kb)

Additional file 6: **Figure S5.** Freshly dissociated tissues were maintained three days in serum-free stem cell-isolating medium supplemented with Epidermal Growth Factor (EGF) and basic Fibroblast Growth Factor (b-FGF). On the left a representative image of the sorting of EpCAM+/CD146+/CD44+ populations (EpCAM+/CD146+/CD44+) and triple negative (EpCAM-/CD146-/CD44-) by FACS ARIA cytometer was reported. Images of colonies of both sorted sub-populations were reported on the right. Yellow and pink boxes mirror cytometer density plot. Pink dashed line represents matrigel front of cell invasion. (PDF 179 kb)

Additional file 7: **Figure S6.** (A) Freshly dissociated tissues maintained for one week in serum-free stem cell-isolating medium supplemented with Epidermal Growth Factor (EGF), basic Fibroblast Growth Factor (b-FGF), DMEM (Dulbecco Modified Eagle Medium), or Glutamine and FBS (Fetal Bovine Serum) supplemented medium, and analyzed by cytofluorimetric analysis. CD45 (PE-Cy7), CD146 (PE), CD44 (H450-Pacific Blue) and EpCAM (FITC) antigens were analyzed. TOPRO3 was used for gating vital cells. (B) The histograms report growth rate fold change of cells described in A, 4 and 10 days after sorting. Control represents (red dashed line) value = 1 i.e. reference relative count at sorting and plating day. Mean of three independent experiments is reported. Values are mean ± s.d (C) Colony forming assay of EpCAM+/CD146+/CD44+ and triple negative sorted cells and non-sorted population maintained in culture one week in stem serum free medium (D) Mean colony size of EpCAM+/CD146+/CD44+ and triple negative sorted cells and non-sorted population maintained in culture one week in stem serum free medium. Mean of three independent experiments is reported. Values are mean ± s.d. (PDF 231 kb)

Additional file 8: **Table S2.** Clinical features of 20 collected ccRCC patients including: 2 G1; 7 G2; 8 G3 and 3 G4 processed by RPPA. (PDF 488 kb)

Additional file 9: **Table S3.** List of total and phosphorylated proteins analyzed by RPPA. In blue, positively expressed proteins. (PDF 87 kb)

Additional file 10: **Figure S7.** (A) Two-way unsupervised hierarchical clustering of RPPA data of 2 G1, 7 G2, 8 G3, and 3 G4 ccRCC populations for the expression of stem cell markers reported as heatmap. (B) mRNA expression of *SOX2* gene in G2, G3, and G4 ccRCC samples as assessed by RT-qPCR. Mean of three independent experiments is reported. RRN18S was used as endogenous control. *p < 0.05 (C) Representative immunofluorescence staining of tumor derived spheroids showing positivity for SOX2 (red). Merge is the over-lapping of SOX2 and DAPI staining. Confocal-microscope used Olympus, Fluoview FV1000 (Tokyo, Japan, http://www.olympus-global.com), magnification 40X. (PDF 246 kb)

Additional file 11: **Figure S8.** (A) Representative images of Western blot analysis of pAKT S473 and pERK T202/Y204 proteins in clear renal cancer isolated cells (G3 and G4) and commercial lines [primary tumor 786–0 (786) and metastatic Caki-1 (CK1)]. GAPDH expression was used as internal control (B) Anti-mTOR and VEGFR2 protein staining in two representative samples classified by (ISUP) grading as G3 and G4 cases by immunohistochemistry assay. Microscope used Nikon Eclipse 55i, magnification 20X. (C) Representative images of Western blot analysis of pAKT S473 and pERK T202/Y204 proteins in non-sorted stem serum free clear renal cancer enriched cells (Stem Tot.), EpCAM+/CD146+/CD44+ (Stem+) and triple negative (Stem-) sorted cells vs non-sorted clear renal cancer cells maintained in DMEM-FBS condition (DMEM Tot) and evaluated one week after culture. (D) Two-way unsupervised hierarchical clustering of 18 ccRCC samples for the expression of proteins belonging to the angiogenesis pathway. Highlighted in the yellow box are overexpressed protein commonly shared in samples of patients that underwent progression (red arrows). N1 and M1 samples were excluded from the analysis (E) Receiver operating characteristic (ROC) curve showing sensitivity and specificity of HIF-1 alpha and phospho-mTOR (S2448) protein RPPA expressions in predicting progression. The true positive rate (sensitivity) is plotted in function of the false positive rate (100-specificity). The area under the ROC curve (AUC) represents a measure of how well the HIF-1 alpha and phospho-mTOR (S2448) protein RPPA expressions distinguishes progression group from no progression [0.96 (*p* < 0.001) and 0.87 (*p* = 0.002), respectively] (PDF 197 kb)

Additional file 12: **Table S4.** Clinical features of 30 collected ccRCC patients and used for in vivo models including: 3 G1, 7 G2, 13 G3 and 7 G4. (PDF 456 kb)

Additional file 13: **Figure S9.** Hematoxylin and Eosin staining of PDXs versus parental primary tumor. G4 tumors often retain both epithelial and sarcomatoid phenotypes. Xenografts are frequently representative of most aggressive parental part. Representative images report epithelial and sarcomatoid phenotype belonging to the same patient. Xenograft image mirrors parental tumor aggressive phenotype. (PDF 114 kb)

Additional file 14: **Figure S10.** (A) Kaplan-Meier curve for the Disease Free Survival (DFS) in the engrafted (Engraf) and not engrafted (No Engraf) groups. (B) Histogram showing FACS analysis results of a PDX derived from a G4 metastatic patient with a sarcomatoid phenotype at *esordium* for the expression of selected markers. (C) Luciferase analysis representative image of G4 ccRCC injected mice (seven mice/group) and after 21 days of treatment with Sunitinib by IVIS imaging. (D) Histogram showing luciferase photon emission of the vehicle and Sunitinib treated mice at days 0 and 21 of treatment and evaluated by IVIS imaging. (E) Representative Haematoxylin and Eosin staining of tumor sections from treated mice. Microscope used Nikon Eclipse 55i, magnification 10X (upper panel) and 20X (lower panel) (F) Two-way unsupervised hierarchical clustering of 20 samples for the expression of endpoints representing the most common drug targets. Green arrows represent recurrent patients, while the black arrow represents a metastatic (M1) sample at esordium. (PDF 542 kb)

## Abbreviations

ccRCC: Clear cell RCC
PDX: Patient Derived Xenograft
RCC: Renal Cell Carcinoma
RPPA: Reverse Phase Protein Array
SRC: Sub Renal Capsule
TIC: Tumor initiating cell
