# Supplementary material for: Renal cancer: new models and approach for personalizing therapy
Source: J Exp Clin Cancer Res. 2018 Sep 5;37:217. doi: 10.1186/s13046-018-0874-4 (PMC6126022; doi:10.1186/s13046-018-0874-4)
Supplement: Supplementary file 1 — Table S1. (A) Clinical features of 57 collected ccRCCs patients including: 3 G1; 15 G2; 27 G3 and 12 G4. B. Clinical features of 10 collected ccRCCs patients used for sorting experiments. (ZIP 880 kb) [file 13046_2018_874_MOESM1_ESM.zip › Supplementary Table 1A.pdf]

PATIENT N°	SEX	AGE	G	T	N	M	STAGE	RECURENCE
1	F	79	1	1b	x	0	1	NO
2	M	42	1	1b	x	0	1	NO
3	M	39	1	1b	x	0	1	NO
4	M	38	2	1b	x	0	1	NO
5	F	53	2	1b	x	0	1	NO
6	M	72	2	1b	x	0	1	NO
7	F	64	2	2a	x	0	2	NO
8	M	81	2	2a	x	0	2	NO
9	M	38	2	2b	x	0	2	NO
10	M	62	2	2a	x	0	2	YES
11	M	49	2	1b	x	0	1	NO
12	F	43	2	3a	x	0	3	NO
13	F	45	2	2a	0	0	2	NO
14	F	60	2	3a	x	0	3	NO
15	M	68	2	1b	x	0	1	NO
16	M	47	2	1b	x	0	1	NO
17	F	82	2	1b	x	0	1	NO
18	M	73	2	2a	x	0	2	NO
19	M	65	3	2a	x	0	2	NO
20	M	61	3	1b	x	0	1	YES
21	F	66	3	3a	0	0	3	NO
22	M	44	3	2b	0	0	2	YES
23	M	51	3	1b	x	0	1	NO
24	M	64	3	3a	0	0	3	YES
25	F	60	3	1b	x	0	1	NO
26	M	71	3	3a	0	1	4	-
27	M	52	3	3a	x	0	3	NO
28	M	53	3	3a	x	0	3	NO
29	M	49	3	2a	x	0	2	NO
30	M	73	3	1b	x	0	1	NO
31	F	67	3	1b	x	1	4	-
32	M	83	3	3a	0	1	4	-
33	F	76	3	1b	x	0	1	NO
34	M	62	3	4	0	0	4	YES
35	M	55	3	2b	x	0	2	NO
36	F	72	3	3a	0	0	3	YES
37	M	63	3	3b	0	0	3	YES
38	F	65	3	1b	x	0	1	NO
39	M	68	3	3b	0	0	3	YES
40	F	60	3	3a	x	1	4	-
41	M	68	3	3a	x	0	3	NO
42	M	74	3	1b	x	0	1	YES
43	M	69	3	3a	x	0	3	NO
44	F	42	3	3a	0	0	3	NO
45	M	45	3	2a	x	0	2	NO
46	M	63	4	1b	x	1	4	-
47	M	50	4	3a	0	0	3	YES
48	M	72	4	3a	0	0	3	NO
49	M	63	4	4	0	1	4	-
50	M	82	4	4	1	0	4	NO
51	M	37	4	2a	x	0	2	NO
52	M	52	4	4	1	0	4	NO
53	M	68	4	3a	0	0	3	YES
54	M	64	4	3a	0	0	3	NO
55	M	63	4	4	0	0	4	YES
56	M	75	4	3b	0	0	3	YES
57	M	83	4	1b	x	0	1	YES

Table S1A
