# Supplementary material for: Renal cancer: new models and approach for personalizing therapy
Source: J Exp Clin Cancer Res. 2018 Sep 5;37:217. doi: 10.1186/s13046-018-0874-4 (PMC6126022; doi:10.1186/s13046-018-0874-4)
Supplement: Supplementary file 1 — Table S1. (A) Clinical features of 57 collected ccRCCs patients including: 3 G1; 15 G2; 27 G3 and 12 G4. B. Clinical features of 10 collected ccRCCs patients used for sorting experiments. (ZIP 880 kb) [file 13046_2018_874_MOESM1_ESM.zip › Supplementary Table 1B.pdf]

PATIENT N°	SEX	AGE	G	T	N	M	STAGE
58	F	69	2	1b	x	0	1
59	F	55	2	1b	x	0	1
60	F	68	3	3a	x	0	3
61	F	76	3	3a	0	1	4
62	M	64	3	1b	x	0	1
63	M	81	3	1b	x	0	1
60	M	62	4	3a	x	0	3
65	M	80	4	3a	2	1	4
66	F	50	4	3a	1	1	4
67	M	65	4	3a	x	1	4

Table S1B
