# Supplementary material for: Renal cancer: new models and approach for personalizing therapy
Source: J Exp Clin Cancer Res. 2018 Sep 5;37:217. doi: 10.1186/s13046-018-0874-4 (PMC6126022; doi:10.1186/s13046-018-0874-4)
Supplement: Supplementary file 2 — Figure S1. (A) (PANEL1) Table of 1286 ccRCC patient distribution: 1013 tumor free patients at 36 months from surgery, 130 metastatic (M1) patients at diagnosis time and 143 recurrent patients at 36 months after surgery were reported. (PANEL2) Table of 57 ccRCC cancer patient distribution: 37 tumor free patients at 36 months from surgery, 6 metastatic (M1) patients at diagnosis time and 14 recurrent patients at 36 months from surgery were reported. (B) Representative immunofluorescence of DAPI-stained tumor derived spheroids. (C) Representative image of 7- aminoactinomycin D staining (7AAD) of in vitro isolated populations by flow cytometry. (D) Table reporting distribution of specific antigen expression percentages (%) in all studied ccRCC populations. (E) Representative images of flow cytometry analysis showing the expression of the epithelial and undifferentiated cell markers EpCAM, CD24, CD10, CD90, CD44 and CD146 mesenchymal stem cell markers in ccRCC isolated populations. Background staining was calculated by using appropriate isotype controls. (F) Flow cytometry analysis of cell lines 786–0 and Caki-1 representative of primary and metastatic tumor, respectively. One representative staining of three independent experiments is shown. (PDF 243 kb) [file 13046_2018_874_MOESM2_ESM.pdf]

A

Panel 1

SAMPLES	1286
Tumor free	1013
M1	130
Recurrent patients	143

Panel 2

SAMPLES	57
Tumor free	37
M1	6
Recurrent patients	14

B

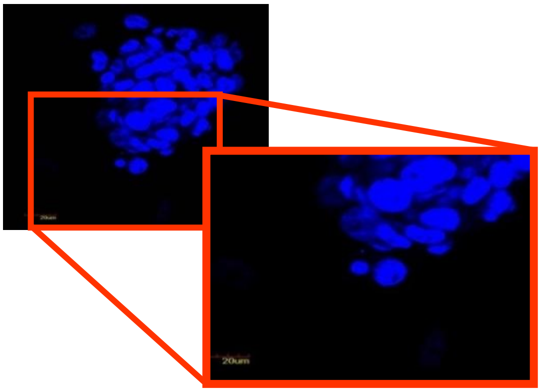

C

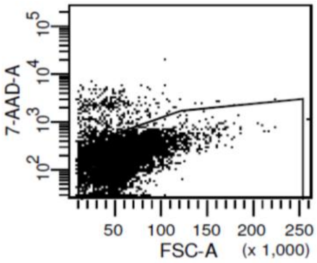

D

CD45	CD90	CD24	CD10
< 10%	<10%	25-60%	1%-20%

CD44	EpCAM	CD146	CK8/18
30-80%	10-70%	1%-30%	40-70%

E

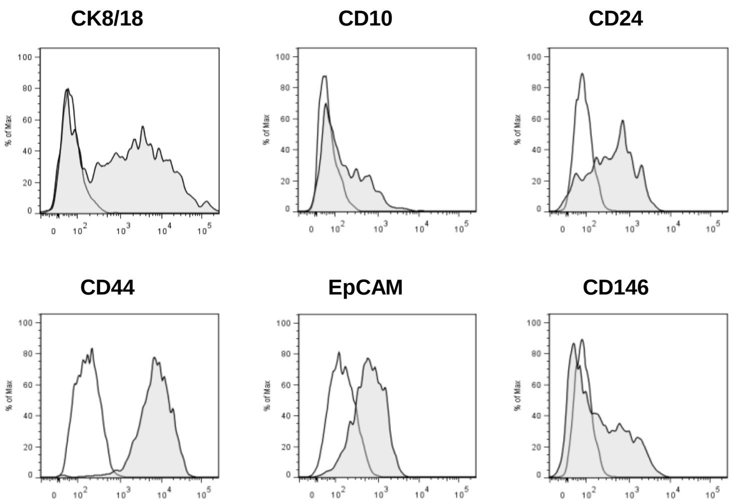

F

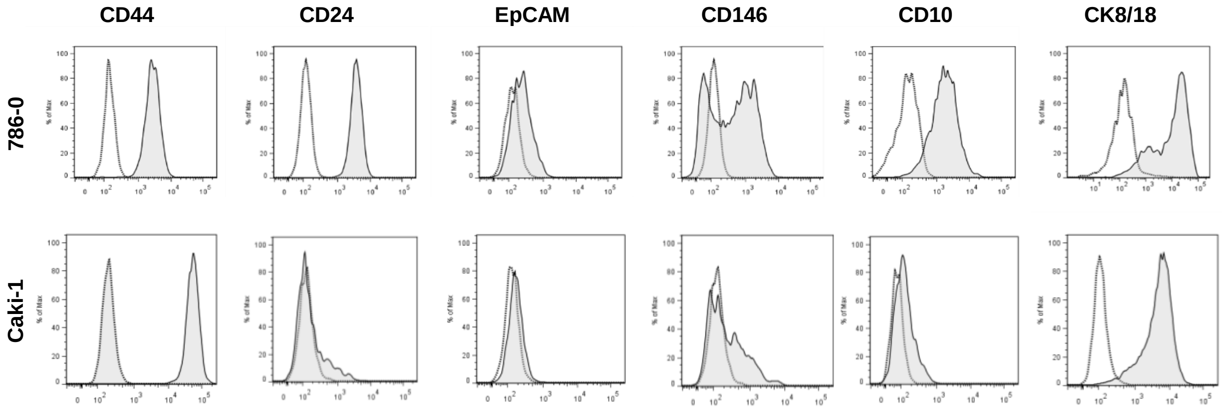

Figure S1
