# Supplementary figures and images for: Renal cancer: new models and approach for personalizing therapy
Source: J Exp Clin Cancer Res. 2018 Sep 5;37:217. doi: 10.1186/s13046-018-0874-4 (PMC6126022; doi:10.1186/s13046-018-0874-4)

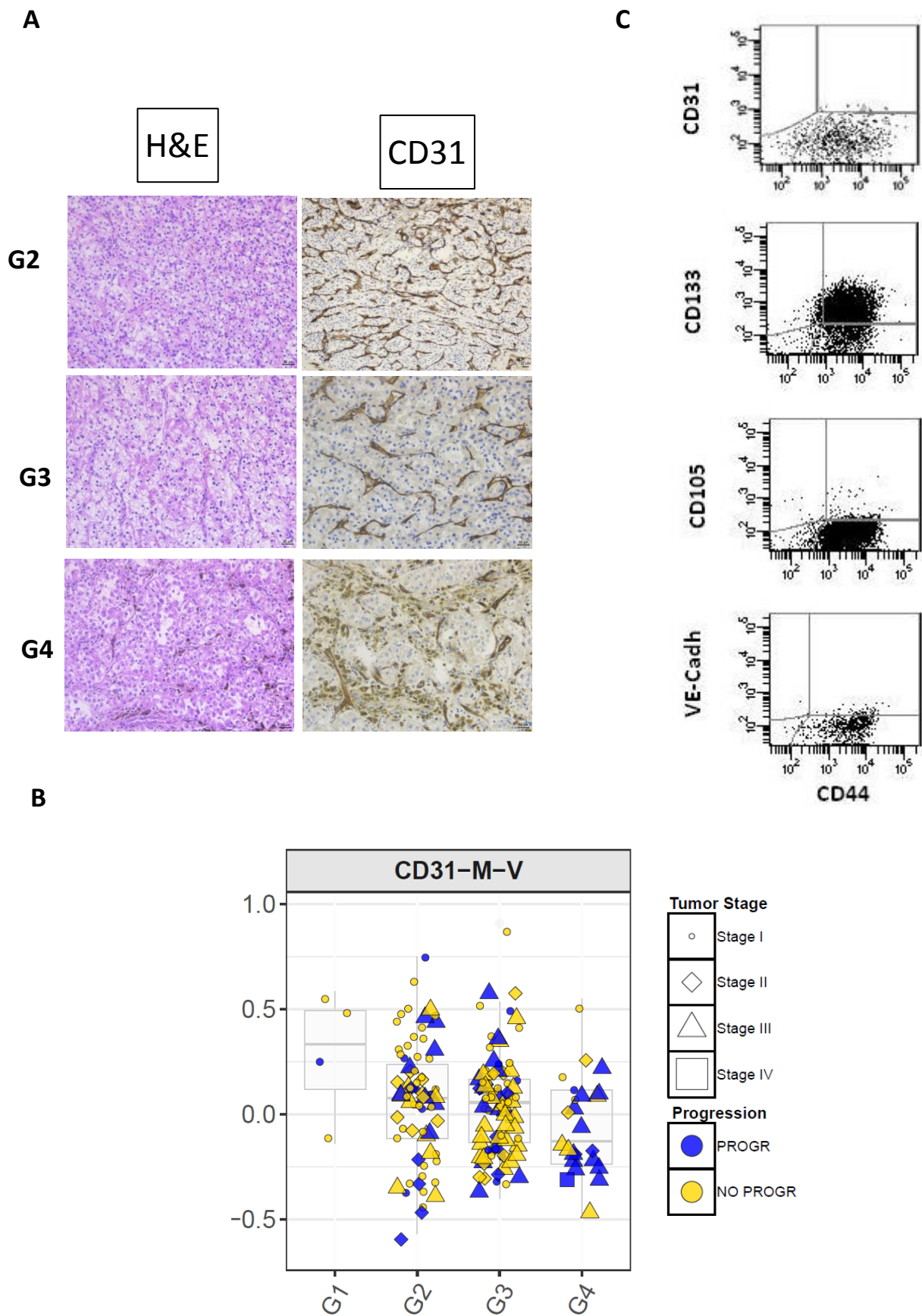

**Figure S2**

Supplement: Supplementary file 3 — Figure S2. (A) Hematoxylin and Eosin (H&E) and CD31 staining of formalin-fixed and paraffin- embedded (FPPE) of primary tumors. Three patients for each grading were analyzed. A representative image for samples is reported. (B) RPPA-TCGA elaboration of CD31 expression. Data were obtained from macrodissected clear cell renal cancer tissues (GDC-database-https://tcga-data.nci.nih.gov/docs/publications/kirc_2013/) and reported for grading, stage and for progression rate by RPPA. (C) Representative images of flow cytometry analysis showing the expression of the endothelial CD31, VE-Cadherin (VE-Cadh) and putative stem cell markers (CD133, CD105) in ccRCC isolated populations. The analysis was combined with CD44 expression. Background staining was calculated by using appropriate isotype controls. (PDF 402 kb) [file 13046_2018_874_MOESM3_ESM.pdf]

# A 3 days of culture after enzymatic dissociation

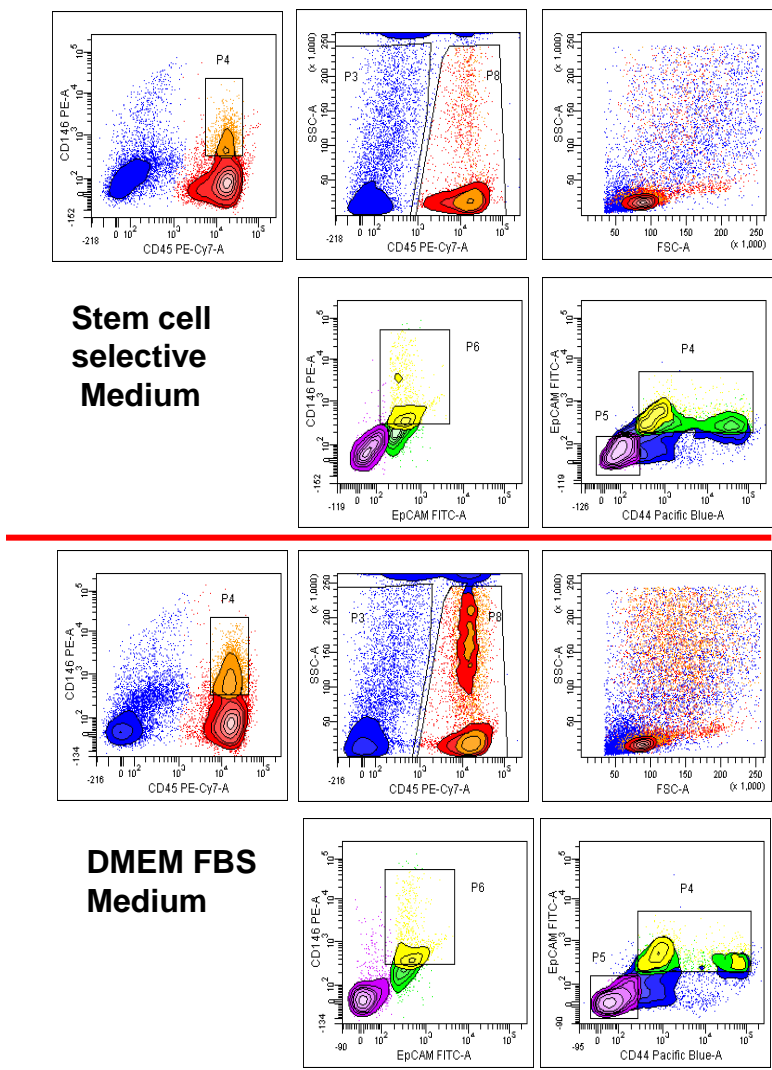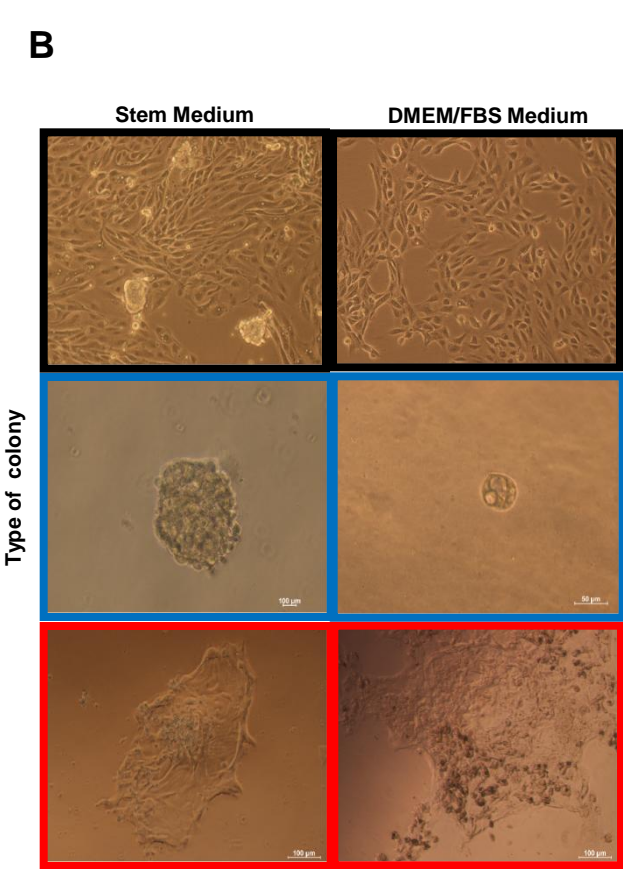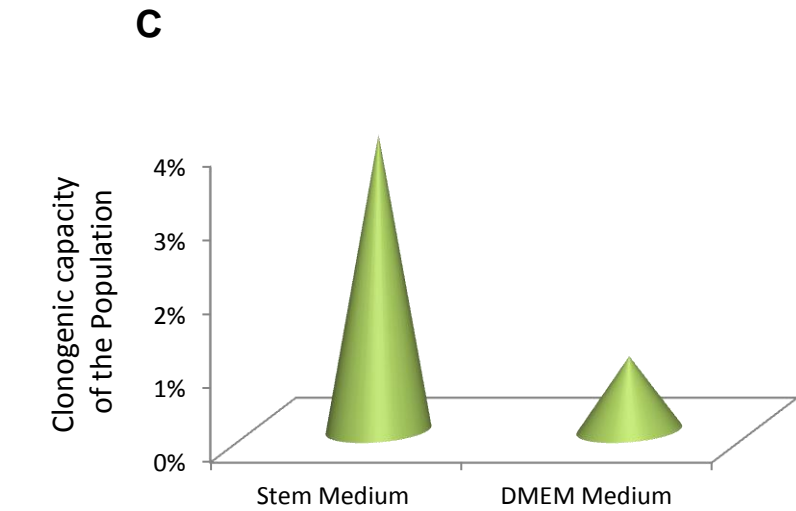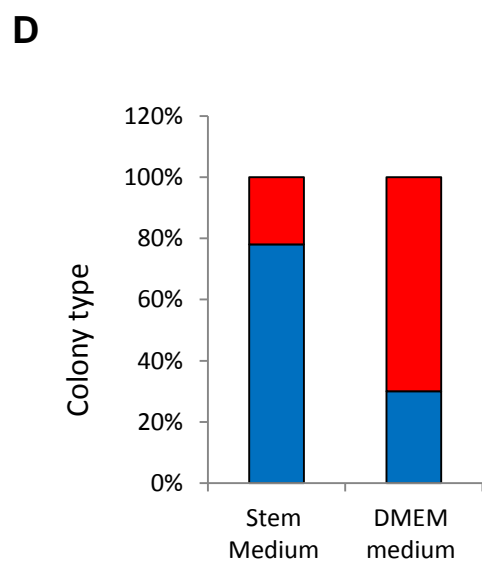

**Figure S4**

Supplement: Supplementary file 5 — Figure S4. (A) Fresh dissociated tissues maintained for three days in serum-free stem cell-isolating medium supplemented with Epidermal Growth Factor (EGF), basic Fibroblast Growth Factor (b-FGF), DMEM (Dulbecco Modified Eagle Medium), or in Glutamine and FBS (Fetal Bovine Serum) supplemented medium, and analyzed by cytofluorimetric analysis. CD45 (PE-Cy7), CD146(PE), CD44 (H450-Pacific Blue) and EpCAM(FITC) antigens were analyzed. TOPRO3 was used for gating vital cells. (B-C) Images and clonogenic population percentage of cells maintained in both conditions after three days of culture by Colony forming assay. Colonies distinguished on the basis of their shape in the two conditions: spheroidal (blue box) and bidimensional (red box). (D) Percentage of colonies distinguished on the basis of their shape in the two conditions was reported: spheroidal (blue box) and bidimentional (red box) such as in B. (PDF 229 kb) [file 13046_2018_874_MOESM5_ESM.pdf]

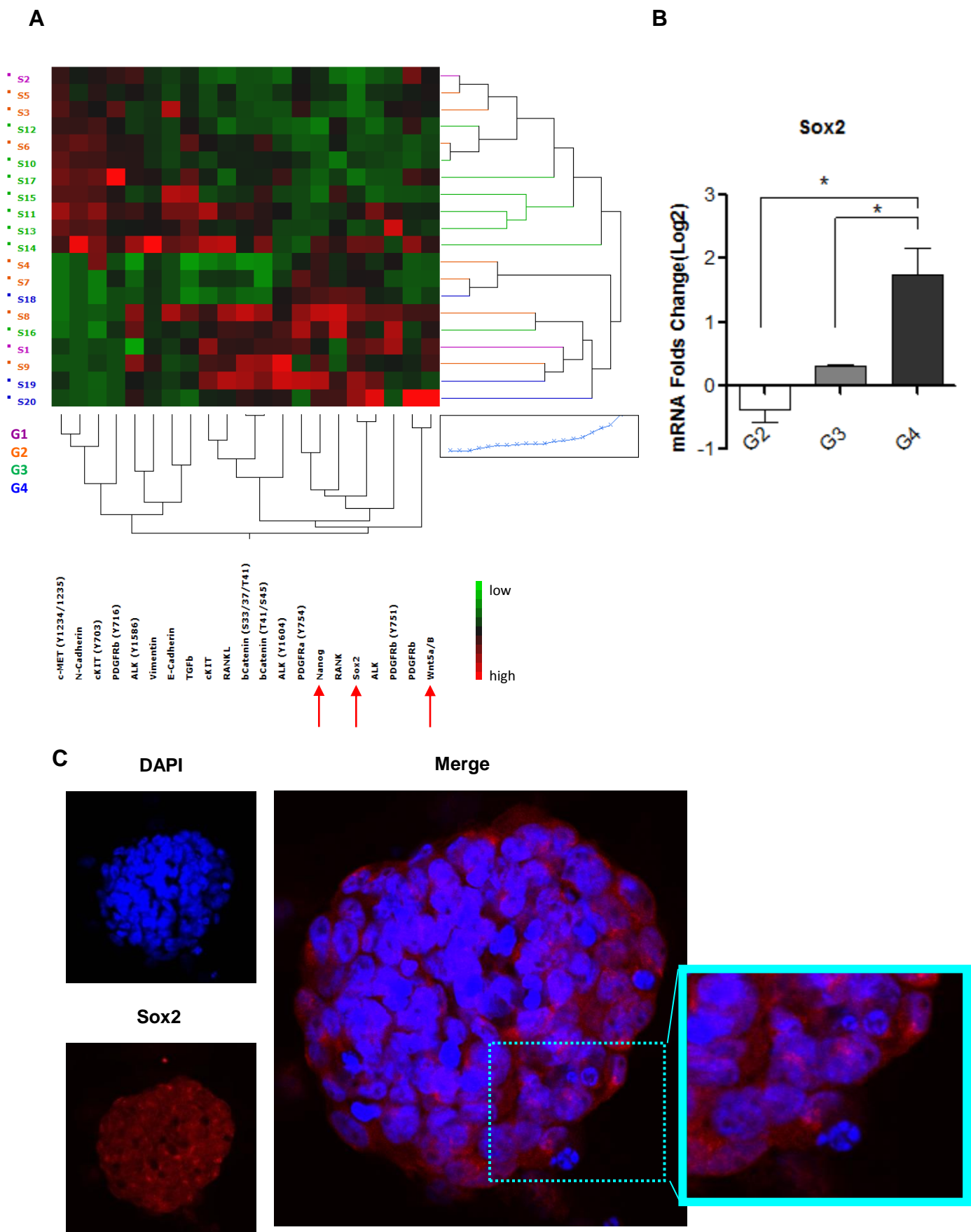

Figure S7

Supplement: Supplementary file 10 — Figure S7. (A) Two-way unsupervised hierarchical clustering of RPPA data of 2 G1, 7 G2, 8 G3, and 3 G4 ccRCC populations for the expression of stem cell markers reported as heatmap. (B) mRNA expression of SOX2 gene in G2, G3, and G4 ccRCC samples as assessed by RT-qPCR. Mean of three independent experiments is reported. RRN18S was used as endogenous control. *p < 0.05 (C) Representative immunofluorescence staining of tumor derived spheroids showing positivity for SOX2 (red). Merge is the over-lapping of SOX2 and DAPI staining. Confocal-microscope used Olympus, Fluoview FV1000 (Tokyo, Japan, http://www.olympus-global.com), magnification 40X. (PDF 246 kb) [file 13046_2018_874_MOESM10_ESM.pdf]

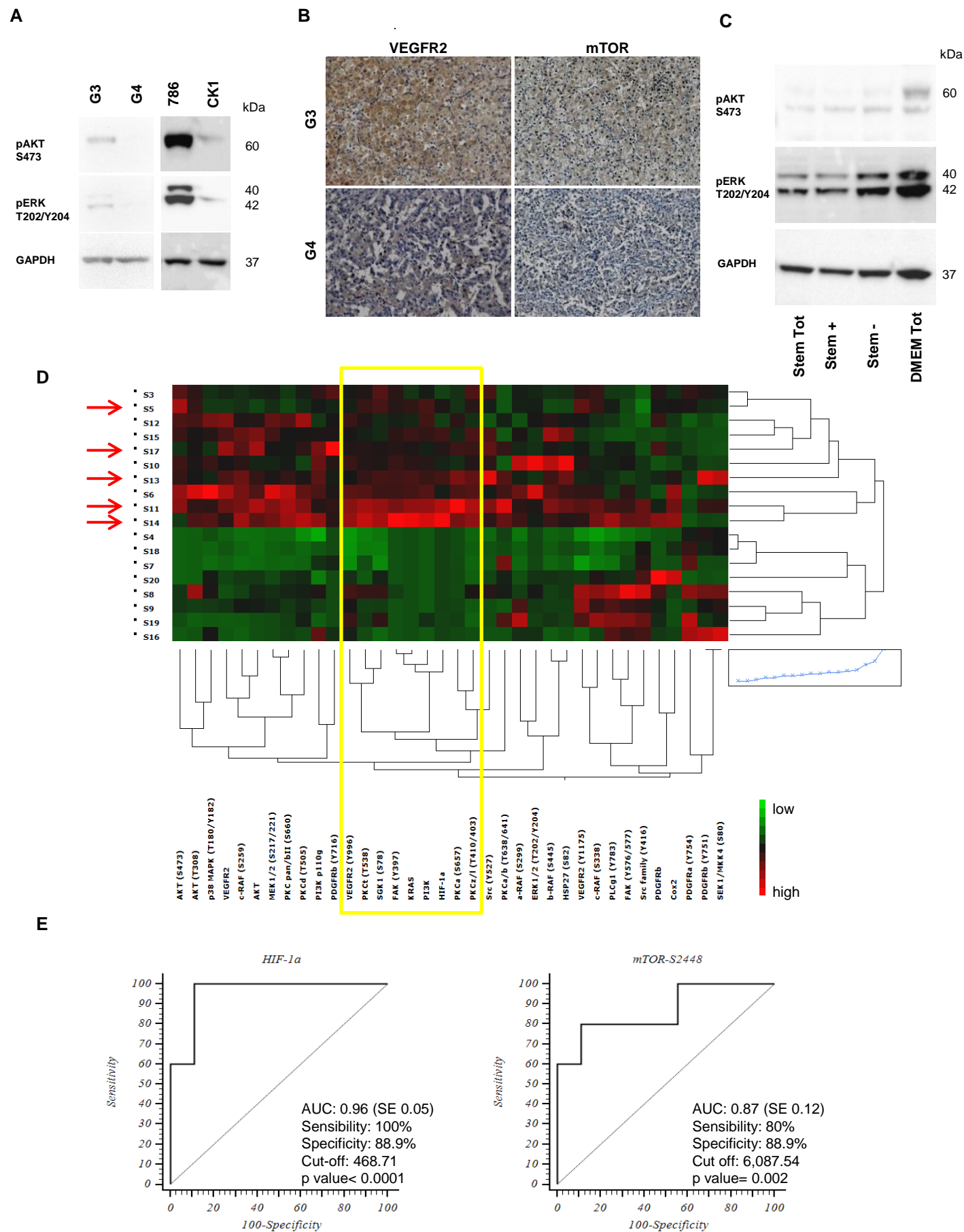

**Figure S8**

Supplement: Supplementary file 11 — Figure S8. (A) Representative images of Western blot analysis of pAKT S473 and pERK T202/Y204 proteins in clear renal cancer isolated cells (G3 and G4) and commercial lines [primary tumor 786–0 (786) and metastatic Caki-1 (CK1)]. GAPDH expression was used as internal control (B) Anti-mTOR and VEGFR2 protein staining in two representative samples classified by (ISUP) grading as G3 and G4 cases by immunohistochemistry assay. Microscope used Nikon Eclipse 55i, magnification 20X. (C) Representative images of Western blot analysis of pAKT S473 and pERK T202/Y204 proteins in non-sorted stem serum free clear renal cancer enriched cells (Stem Tot.), EpCAM+/CD146+/CD44+ (Stem+) and triple negative (Stem-) sorted cells vs non-sorted clear renal cancer cells maintained in DMEM-FBS condition (DMEM Tot) and evaluated one week after culture. (D) Two-way unsupervised hierarchical clustering of 18 ccRCC samples for the expression of proteins belonging to the angiogenesis pathway. Highlighted in the yellow box are overexpressed protein commonly shared in samples of patients that underwent progression (red arrows). N1 and M1 samples were excluded from the analysis (E) Receiver operating characteristic (ROC) curve showing sensitivity and specificity of HIF-1 alpha and phospho-mTOR (S2448) protein RPPA expressions in predicting progression. The true positive rate (sensitivity) is plotted in function of the false positive rate (100-specificity). The area under the ROC curve (AUC) represents a measure of how well the HIF-1 alpha and phospho-mTOR (S2448) protein RPPA expressions distinguishes progression group from no progression [0.96 (p < 0.001) and 0.87 (p = 0.002), respectively] (PDF 197 kb) [file 13046_2018_874_MOESM11_ESM.pdf]

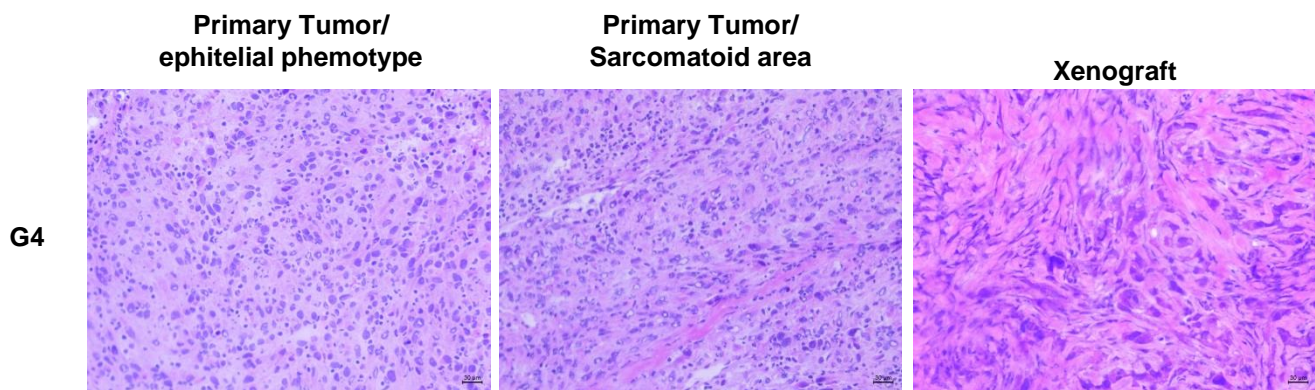

**Figure S9**

Supplement: Supplementary file 13 — Figure S9. Hematoxylin and Eosin staining of PDXs versus parental primary tumor. G4 tumors often retain both epithelial and sarcomatoid phenotypes. Xenografts are frequently representative of most aggressive parental part. Representative images report epithelial and sarcomatoid phenotype belonging to the same patient. Xenograft image mirrors parental tumor aggressive phenotype. (PDF 114 kb) [file 13046_2018_874_MOESM13_ESM.pdf]

A

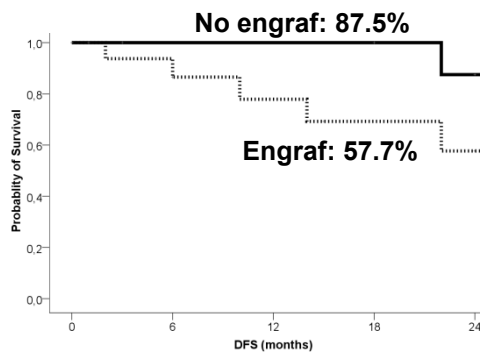

B

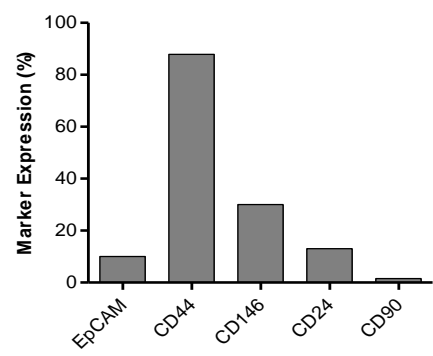

C

After Treatment – day 21

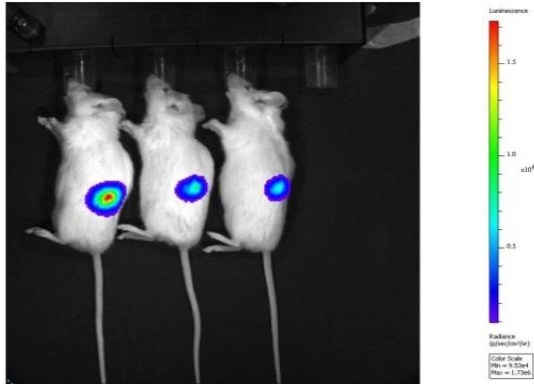

D

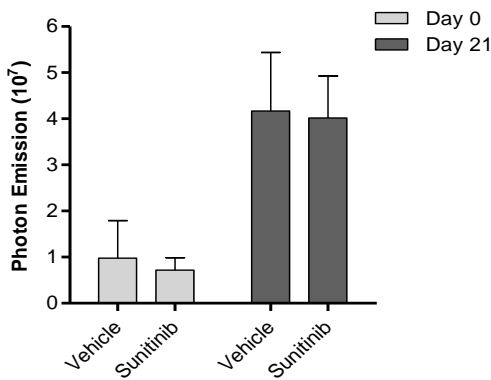

E

After Treatment – day 21

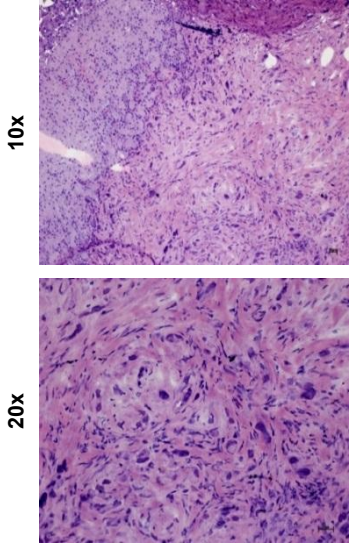

F

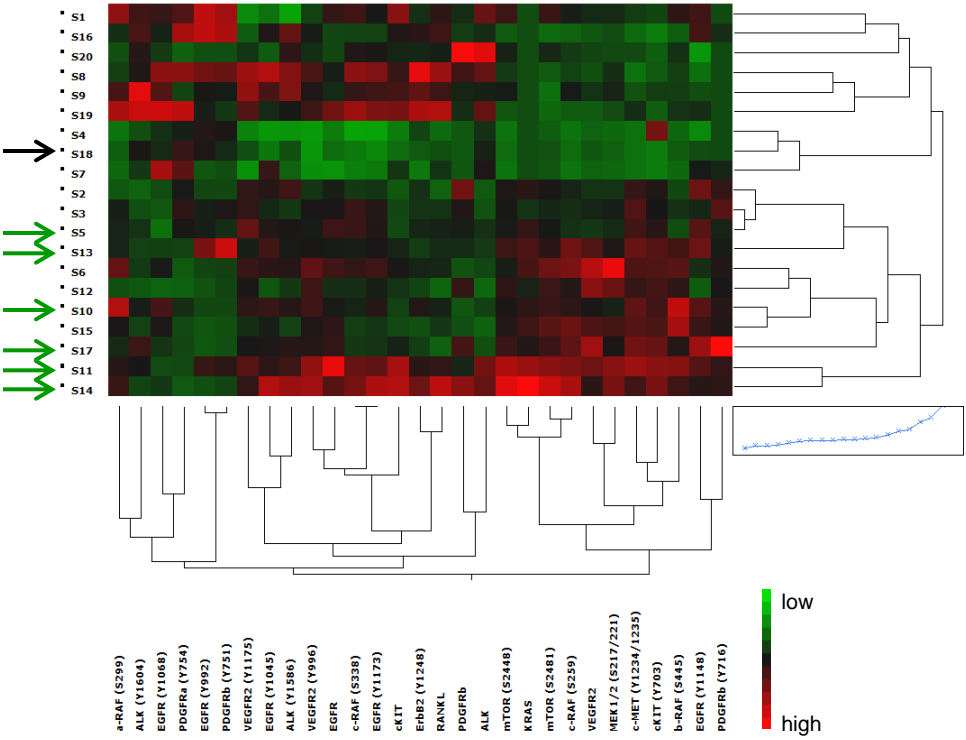

Figure S10

Supplement: Supplementary file 14 — Figure S10. (A) Kaplan-Meier curve for the Disease Free Survival (DFS) in the engrafted (Engraf) and not engrafted (No Engraf) groups. (B) Histogram showing FACS analysis results of a PDX derived from a G4 metastatic patient with a sarcomatoid phenotype at esordium for the expression of selected markers. (C) Luciferase analysis representative image of G4 ccRCC injected mice (seven mice/group) and after 21 days of treatment with Sunitinib by IVIS imaging. (D) Histogram showing luciferase photon emission of the vehicle and Sunitinib treated mice at days 0 and 21 of treatment and evaluated by IVIS imaging. (E) Representative Haematoxylin and Eosin staining of tumor sections from treated mice. Microscope used Nikon Eclipse 55i, magnification 10X (upper panel) and 20X (lower panel) (F) Two-way unsupervised hierarchical clustering of 20 samples for the expression of endpoints representing the most common drug targets. Green arrows represent recurrent patients, while the black arrow represents a metastatic (M1) sample at esordium. (PDF 542 kb) [file 13046_2018_874_MOESM14_ESM.pdf]
