# Supplementary material for: Renal cancer: new models and approach for personalizing therapy
Source: J Exp Clin Cancer Res. 2018 Sep 5;37:217. doi: 10.1186/s13046-018-0874-4 (PMC6126022; doi:10.1186/s13046-018-0874-4)
Supplement: Supplementary file 4 — Figure S3 (A) RPPA-TCGA elaboration of E-Cadherin and Fibronectin expressions. Data were obtained from macrodissected clear cell renal cancer tissues (GDC-database-https://tcga-data.nci.nih.gov/docs/publications/kirc_2013/) and reported for grading, stage and for progression rate by RPPA. (B) mRNA level elaboration of EpCAM, CD146(MCAM) and CD44 antigens. Data were obtained from GSE48550 microarray and were analyzed on different kinds of renal stem cells. (C) TOPRO3 staining for cell viability evaluation of populations maintained for three days (upper panels) and one week (Lower panels) in serum-free stem cell-isolating medium supplemented with Epidermal Growth Factor (EGF), basic Fibroblast Growth Factor (b-FGF), DMEM (Dulbecco Modified Eagle Medium), Glutamine and FBS (Fetal Bovine Serum) supplemented medium and evaluated by cytofluorimetric analysis. Blue and Black areas represent vital and dead cells respectively. (PDF 389 kb) [file 13046_2018_874_MOESM4_ESM.pdf]

A

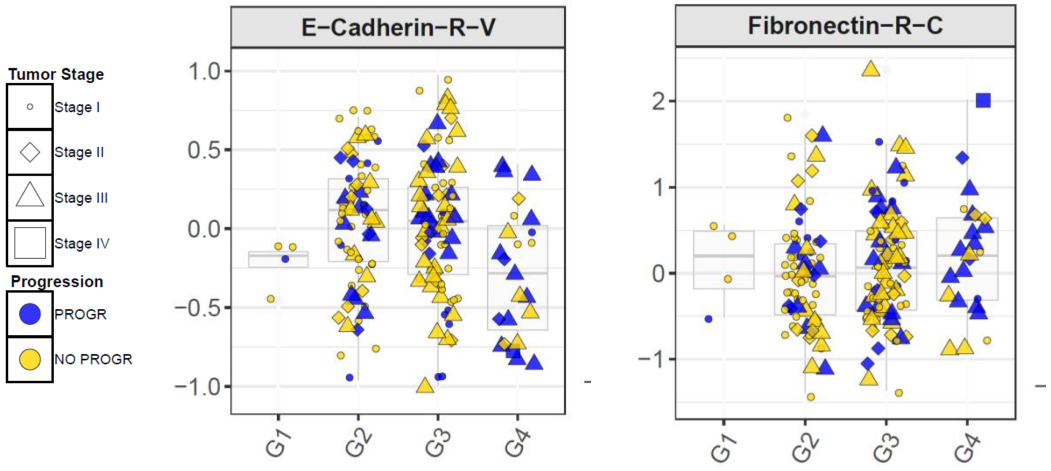

B

GSE48550 microarray data-gene selection (mRNA)

Data from *Galleggiante V. Et al. The Journal of Urology. 2014*

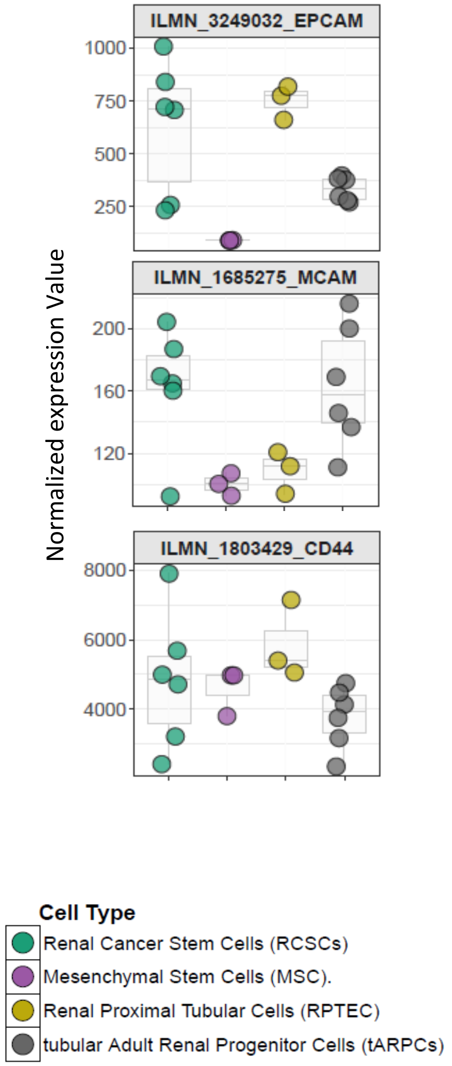

C

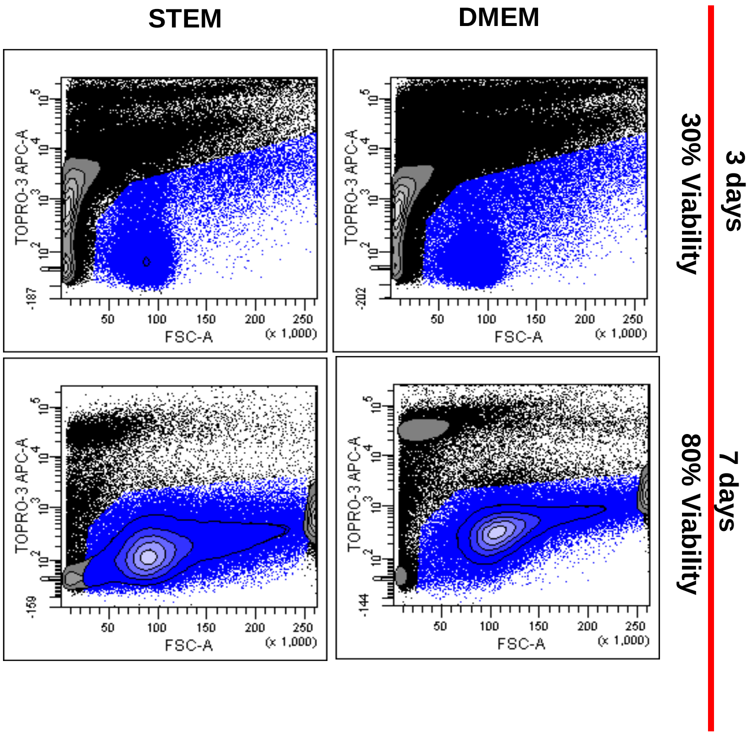

Figure S3
