# Supplementary material for: Renal cancer: new models and approach for personalizing therapy
Source: J Exp Clin Cancer Res. 2018 Sep 5;37:217. doi: 10.1186/s13046-018-0874-4 (PMC6126022; doi:10.1186/s13046-018-0874-4)
Supplement: Supplementary file 6 — Figure S5. Freshly dissociated tissues were maintained three days in serum-free stem cell-isolating medium supplemented with Epidermal Growth Factor (EGF) and basic Fibroblast Growth Factor (b-FGF). On the left a representative image of the sorting of EpCAM+/CD146+/CD44+ populations (EpCAM+/CD146+/CD44+) and triple negative (EpCAM-/CD146-/CD44-) by FACS ARIA cytometer was reported. Images of colonies of both sorted sub-populations were reported on the right. Yellow and pink boxes mirror cytometer density plot. Pink dashed line represents matrigel front of cell invasion. (PDF 179 kb) [file 13046_2018_874_MOESM6_ESM.pdf]

Stem cell selective Medium

3 day after enzymatic dissociation Post-Sorting-clonogenesis

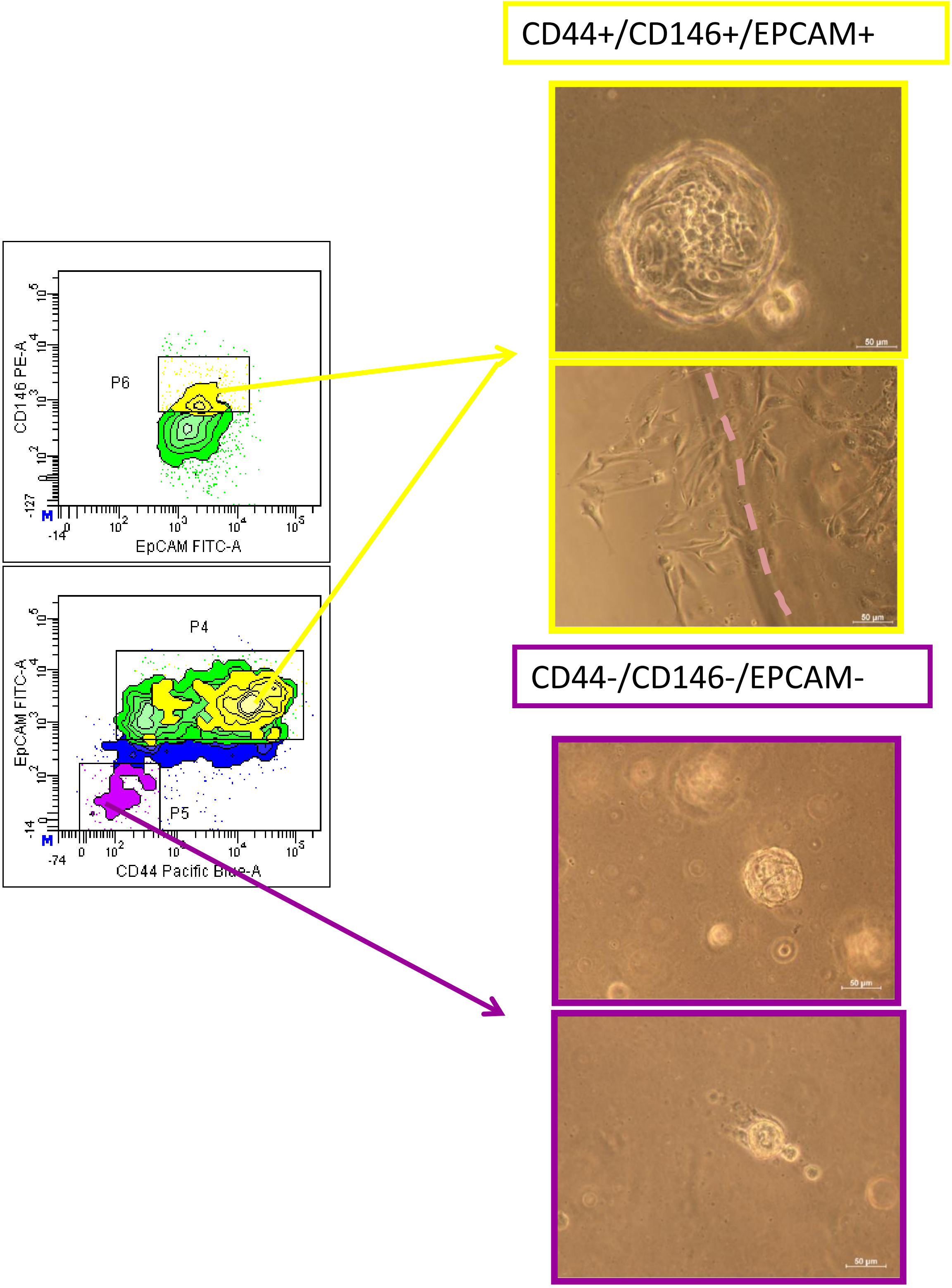

Figure S5
