# Supplementary material for: Renal cancer: new models and approach for personalizing therapy
Source: J Exp Clin Cancer Res. 2018 Sep 5;37:217. doi: 10.1186/s13046-018-0874-4 (PMC6126022; doi:10.1186/s13046-018-0874-4)
Supplement: Supplementary file 7 — Figure S6. (A) Freshly dissociated tissues maintained for one week in serum-free stem cell-isolating medium supplemented with Epidermal Growth Factor (EGF), basic Fibroblast Growth Factor (b-FGF), DMEM (Dulbecco Modified Eagle Medium), or Glutamine and FBS (Fetal Bovine Serum) supplemented medium, and analyzed by cytofluorimetric analysis. CD45 (PE-Cy7), CD146 (PE), CD44 (H450-Pacific Blue) and EpCAM (FITC) antigens were analyzed. TOPRO3 was used for gating vital cells. (B) The histograms report growth rate fold change of cells described in A, 4 and 10 days after sorting. Control represents (red dashed line) value = 1 i.e. reference relative count at sorting and plating day. Mean of three independent experiments is reported. Values are mean ± s.d (C) Colony forming assay of EpCAM+/CD146+/CD44+ and triple negative sorted cells and non-sorted population maintained in culture one week in stem serum free medium (D) Mean colony size of EpCAM+/CD146+/CD44+ and triple negative sorted cells and non-sorted population maintained in culture one week in stem serum free medium. Mean of three independent experiments is reported. Values are mean ± s.d. (PDF 231 kb) [file 13046_2018_874_MOESM7_ESM.pdf]

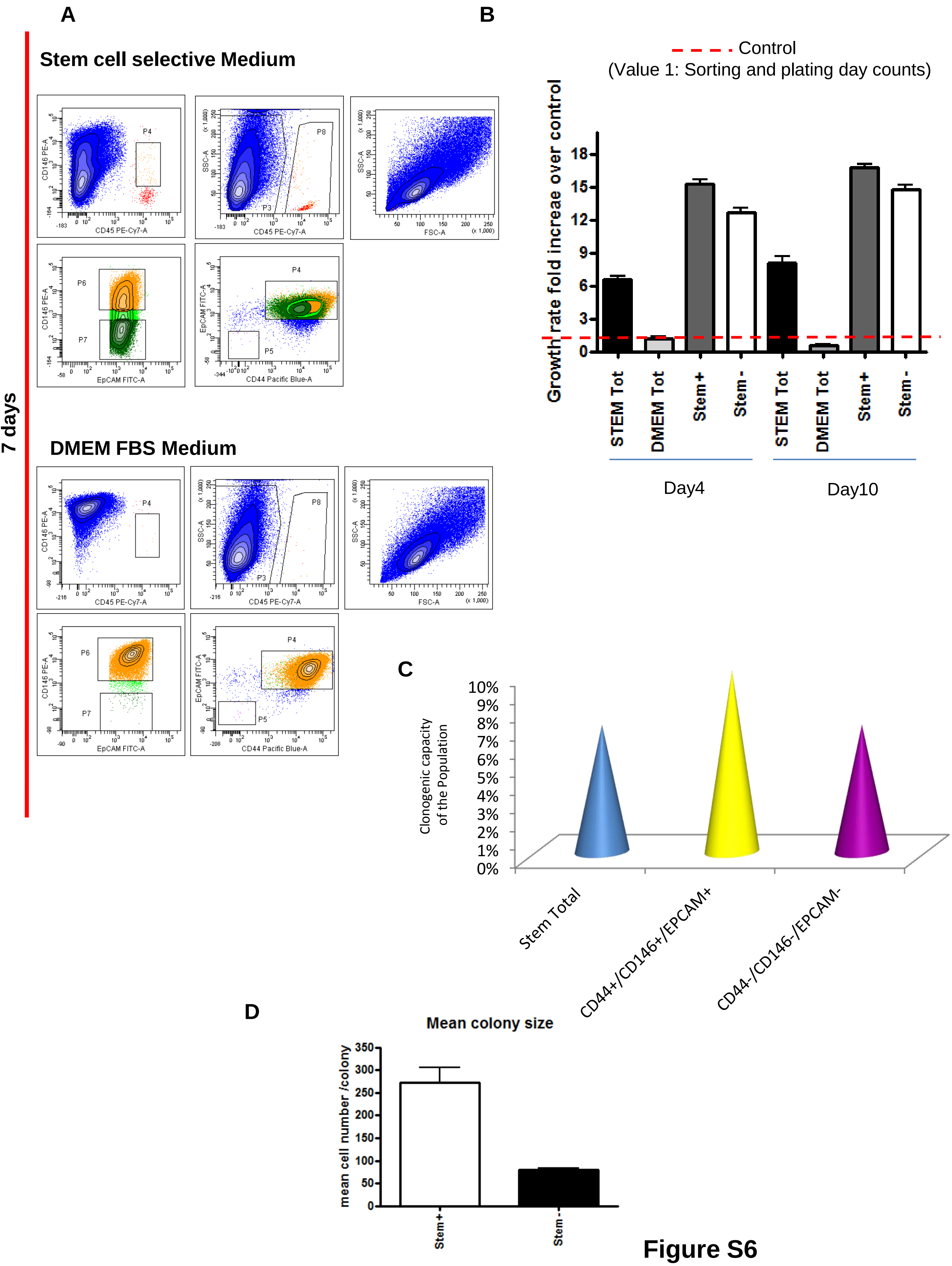
A
B
7 days
Stem cell selective Medium
DMEM FBS Medium
CD146 PE-A
CD45 PE-Cy7-A
SSC-A
FSC-A
EpCAM FITC-A
CD44 Pacific Blue-A
P3
P4
P5
P6
P7
P8
Control
(Value 1: Sorting and plating day counts)
Growth rate fold increae over control
STEM Tot
DMEM Tot
Stem+
Stem -
Day4
Day10
C
Clonogenic capacity of the Population
Stem Total
CD44+/CD146+/EPCAM+
CD44-/CD146-/EPCAM-
D
Mean colony size
mean cell number /colony
Stem+
Stem-

Figure S6
