# Supplementary material for: Renal cancer: new models and approach for personalizing therapy
Source: J Exp Clin Cancer Res. 2018 Sep 5;37:217. doi: 10.1186/s13046-018-0874-4 (PMC6126022; doi:10.1186/s13046-018-0874-4)
Supplement: Supplementary file 8 — Table S2. Clinical features of 20 collected ccRCC patients including: 2 G1; 7 G2; 8 G3 and 3 G4 processed by RPPA. (PDF 488 kb) [file 13046_2018_874_MOESM8_ESM.pdf]

SAMPLE N°	SEX	AGE	G	T	N	M	STAGE
S1	F	79	1	1b	x	0	1
S2	M	39	1	1b	x	0	1
S3	F	53	2	1b	x	0	1
S4	M	38	2	2b	x	0	2
S5	M	62	2	2a	x	0	2
S6	M	49	2	1b	x	0	1
S7	F	43	2	3a	x	0	3
S8	F	45	2	2a	0	0	2
S9	M	68	2	1b	x	0	1
S10	M	64	3	3a	0	0	3
S11	M	62	3	4	0	0	4
S12	M	55	3	2b	x	0	2
S13	F	72	3	3a	0	0	3
S14	M	63	3	3b	0	0	3
S15	F	65	3	1b	x	0	1
S16	M	68	3	3a	x	0	3
S17	M	74	3	1b	x	0	1
S18	M	63	4	4	0	1	4
S19	M	82	4	4	1	0	4
S20	M	37	4	2a	x	0	2

Table S2
