# Supplementary material for: Renal cancer: new models and approach for personalizing therapy
Source: J Exp Clin Cancer Res. 2018 Sep 5;37:217. doi: 10.1186/s13046-018-0874-4 (PMC6126022; doi:10.1186/s13046-018-0874-4)
Supplement: Supplementary file 9 — Table S3. List of total and phosphorylated proteins analyzed by RPPA. In blue, positively expressed proteins. (PDF 87 kb) [file 13046_2018_874_MOESM9_ESM.pdf]

ENDPOINTS ANALYZED BY RPPA		
4E-BP1 (S65)	EGFR (Y1068)	p90RSK (S380)
4E-BP1 (T37/46)	EGFR (Y1148)	Paxillin (Y118)
4E-BP1 (T70)	EGFR (Y1173)	PDGFRα (Y754)
AKT	EGFR (Y992)	PDGFRβ
AKT (S473)	eIF4E (S209)	PDGFRβ (Y716)
AKT (T308)	eIF4G (S1108)	PDGFRβ (Y751)
ALK	Elk-1 (S383)	PDK1 (S241)
ALK (Y1586)	ErbB2 (Y1248)	PI3K
ALK (Y1604)	ErbB3	PI3Kp110γ
AMPKα (S485)	ErbB3 (Y1289)	PKCpan/βII (S660)
AMPKβ (S108)	ErbB4	PKCα (S657)
a-RAF (S299)	ERG	PKCα/β (T638/641)
Ask1 (S83)	ERK1/2 (T202/Y204)	PKCδ (T505)
Aurora A (T288)/B (T232)/C (T198)	Ezrin (T567)/Radixin (T564)/Moesin (T558)	PKCζ/λ (T410/403)
Aurora A/AIK	FAK (Y397)	PKCθ (T538)
βCatenin (S33/37/T41)	FAK (Y576/577)	PLCγ1 (Y783)
βCatenin (T41/S45)	GSK-3α/β (S21/9)	PRAS40 (T246)
BAD	GSK-3α/β (S279/216)	RANK
BAD (S112)	HIF-1α	RANKL
BAD (S136)	HSP27 (S82)	RSK3 (T356/S360)
BAK	HSP70	S6 Ribosomal Protein (S235/236)
BAX	HSP90a (T5/7)	S6 Ribosomal Protein (S240/244)
Bcl-2 (S70)	IGF-1R (Y1131)/IR (Y1146)	SEK1/MKK4 (S80)
Bcl-2 (T56)	IGF-1R (Y1135/1136)/IR (Y1150/1151)	SGK1 (S78)
Bcl-xL	IkBα (S32/36)	Sox2
b-RAF (S445)	IL-6	Src (Y527)
cKIT	IRS-1 (S612)	Src family (Y416)
cKIT (Y703)	KRAS	STAT3
c-Myc	MARCKS (S152/156)	STAT3 (S727)
c-MET (Y1234/1235)	MDM2 (S166)	STAT3 (Y705)
c-RAF (S259)	MEK1/2 (S217/221)	STAT5
c-RAF (S338)	mTOR (S2448)	STAT5 (Y694)
Cofilin (S3)	mTOR (S2481)	Survivin
Cox2	Nanog	TGFβ
CrkII (Y221)	N-Cadherin	Tuberin/TSC2 (Y1571)
CrkL (Y207)	p38 MAPK (T180/Y182)	VEGFR2
Cyclin D1	p53 (S15)	VEGFR2 (Y1175)
E-Cadherin	p53 (S46)	VEGFR2 (Y951)
EGFR	p70 S6K (S371)	VEGFR2 (Y996)
EGFR (S1046/1047)	p70 S6K (T389)	Vimentin
EGFR (Y1045)	p70 S6K (T412)	Wnt5a/b

Table S3
