# Supplementary material for: Renal cancer: new models and approach for personalizing therapy
Source: J Exp Clin Cancer Res. 2018 Sep 5;37:217. doi: 10.1186/s13046-018-0874-4 (PMC6126022; doi:10.1186/s13046-018-0874-4)
Supplement: Supplementary file 12 — Table S4. Clinical features of 30 collected ccRCC patients and used for in vivo models including: 3 G1, 7 G2, 13 G3 and 7 G4. (PDF 456 kb) [file 13046_2018_874_MOESM12_ESM.pdf]

PDX N°	SEX	G	T	N	M	RECURENCE	TUMORGRAFTS
1	F	1	1b	x	0	NO	NO
2	M	1	1b	x	0	NO	NO
3	M	1	1b	x	0	NO	NO
4	M	2	1b	x	0	NO	NO
5	M	2	2a	x	0	YES	YES
6	M	2	1b	x	0	NO	NO
7	F	2	3a	x	0	NO	NO
8	F	2	2a	0	0	NO	YES
9	M	2	1b	x	0	NO	NO
10	M	2	2a	x	0	NO	YES
11	F	3	3a	0	0	NO	YES
12	M	3	1b	x	0	NO	YES
13	M	3	3a	0	0	YES	NO
14	F	3	1b	x	0	NO	NO
15	M	3	3a	x	0	NO	NO
16	F	3	1b	x	0	NO	NO
17	M	3	4	0	0	YES	YES
18	M	3	2b	x	0	NO	YES
19	F	3	3a	0	0	YES	YES
20	F	3	1b	x	0	NO	YES
21	M	3	3b	0	0	YES	YES
22	F	3	3a	x	1	-	YES
23	M	3	1b	x	0	YES	YES
24	M	4	1b	x	1	-	YES
25	M	4	3a	0	0	YES	NO
26	M	4	4	0	1	-	YES
27	M	4	4	1	0	NO	YES
28	M	4	2a	x	0	NO	YES
29	M	4	4	0	0	YES	YES
30	M	4	1b	x	0	YES	YES

Table S4
